# Raman Spectroscopy Study of Structurally Uniform Hydrogenated Oligomers of α-Olefins

**DOI:** 10.3390/polym12092153

**Published:** 2020-09-21

**Authors:** Sergey M. Kuznetsov, Maria S. Iablochnikova, Elena A. Sagitova, Kirill A. Prokhorov, Gulnara Yu. Nikolaeva, Leila Yu. Ustynyuk, Pavel V. Ivchenko, Alexey A. Vinogradov, Alexander A. Vinogradov, Ilya E. Nifant’ev

**Affiliations:** 1 Prokhorov General Physics Institute of the Russian Academy of Sciences, Vavilov Str. 38, 119991 Moscow, Russia; kuznetsovsm@kapella.gpi.ru (S.M.K.); iablochnikova.ms@phystech.edu (M.S.I.); homa1971@gmail.com (G.Y.N.); 2 Moscow Institute of Physics and Technology, Institutskiy Per. 9, 141700 Dolgoprudnyi, Moscow Region, Russia; 3 Department of Chemistry, M.V. Lomonosov Moscow State University, Leninskie Gory 1-3, 119991 Moscow, Russia; leila_ust@mail.ru (L.Y.U.); inpv@org.chem.msu.ru (P.V.I.); inif@org.chem.msu.ru (I.E.N.); 4 A.V. Topchiev Institute of Petrochemical Synthesis of the Russian Academy of Sciences, Leninsky Pr. 29, 119991 Moscow, Russia; vinasora@ips.ac.ru (A.A.V.); amvin@mail.ru (A.A.V.)

**Keywords:** α-olefin oligomers, hydrocarbon conformations, DFT, polyolefins, Raman spectroscopy

## Abstract

The expansion of the range of physico-chemical methods in the study of industrially significant α-olefin oligomers and polymers is of particular interest. In our article, we present a comparative Raman study of structurally uniform hydrogenated dimers, trimers, tetramers, and pentamers of 1-hexene and 1-octene, that are attractive as bases for freeze-resistant engine oils and lubricants. We found out that the joint monitoring of the disorder longitudinal acoustic mode (D-LAM) and symmetric C–C stretching modes allows the quantitative characterization of the number and length of alkyl chains (i.e., two structural characteristics), upon which the pour point and viscosity of the hydrocarbons depend, and to distinguish these compounds from both each other and linear alkanes. We demonstrated that the ratio of the contents of CH_2_ and CH_3_ groups in these hydrocarbons can be determined by using the intensities of the bands in the spectra, related to the asymmetric stretching vibrations of these groups. The density functional theory (DFT) calculations were applied to reveal the relations between the wavenumber and bandshape of the symmetric C–C stretching mode and a conformation arrangement of the 1-hexene and 1-octene dimers. We found that the branched double-chain conformation results in the splitting of the C–C mode into two components with the wavenumbers, which can be used as a measure of the length of branches. This conformation is preferable to the extended-chain conformation for hydrogenated 1-hexene and 1-octene dimers.

## 1. Introduction

**Figure 1:**
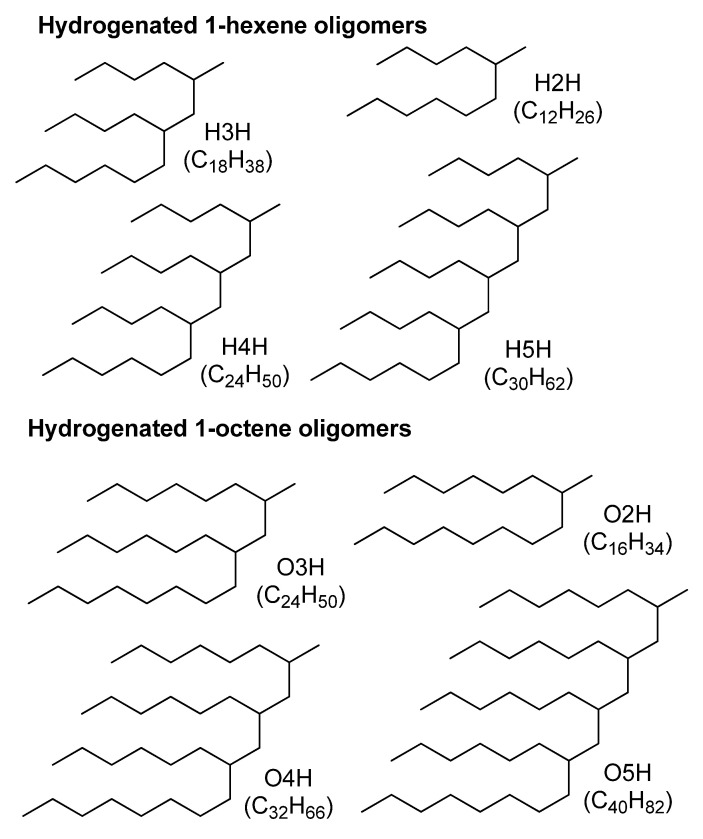
The chemical formulae of the hydrogenated 1-hexene and 1-octene oligomers: dimers (H2H and O2H), trimers (H3H and O3H), tetramers (H4H and O4H), pentamers (H5H and O5H).

Recently, Nifant’ev I.E. et al. [1] and then Bahri-Laleh et al. [2] succeeded to synthesize the series of structurally uniform branched alkanes that represent hydrogenated 1-hexene and 1-octene oligomers (dimers, trimers, tetramers, and pentamers, Figure 1). The high structural homogeneity of these compounds was attributable to the use of specific heterocene catalyst, activated with minimal amounts of methylalumoxane [1]. In that works, the authors explored these compounds by different techniques, including ^1^H and ^13^C NMR spectroscopy, gas chromatography, viscosity, and pour point testing to reveal their chemical compositions, pour points, and viscosity–temperature relationships [1,2].

At room temperature, these oligomers are liquids, their molecules contain from two to five alkyl chains, as illustrated in Figure 1. Each chain monomer subunit in the 1-hexene and 1-octene oligomers consists of six and eight carbon atoms, respectively. The total number of the carbon atoms in a molecule varies from 12 to 40. Depending on the number and length of the alkyl chains in a molecule, the pour points and kinematic viscosity at 100 °C (KV^100^) of these liquids vary from –66 to −94 °C; and from 2.2 to 6.5 cSt, respectively. The moderate viscosity at low temperatures and low pour points make these compounds attractive as bases of high-quality freeze-resistant poly-α-olefin oils (PAOs) and lubricants [1,3]. Additionally, such hydrogenated oligomers, like linear alkanes, can be regarded as model structures for studying a molecular arrangement of biomembranes and lipids [4,5,6].

In our previous study of the hydrogenated 1-hexene oligomers [7], we have identified several Raman lines that allow us to determine the length of the alkyl chains and the number of chains in a molecule. These are important structure characteristics that affect the viscosity and pour point of the liquids under study. For example, the viscosity indexes of isomers H4H and O3H differ almost by two times [1]. In this work, for the first time we extend the Raman studies to hydrogenated 1-octene oligomers. Additionally, we compare Raman spectra of the hydrogenated 1-hexene and 1-octene oligomers, as well as linear alkanes, in order to reveal spectral features for distinguishing these substances from each other.

The Raman-active vibrations, whose frequencies depend on a chain length of alkanes and related substances (fatty acids, alkenes, alkylammonium compounds, bromoalkanes, etc.), belong to the longitudinal acoustic mode (LAM), disorder LAM (D-LAM), and symmetric C–C stretching mode [7,8,9,10,11,12,13,14,15,16,17,18,19,20,21].

The LAM is well-known marker to evaluate a length of *trans* and *quasi*-*trans* segments in both linear and branched alkanes [8,9,10,11]. For example, Raman spectroscopic study in the LAM spectral region proved that molecules in the solid-state center-branched alkanes (C_17_H_35_)_2_CHCH_3_ and (C_19_H_39_)_2_CHCH_3_ are not folded and are crystallized as extended chains with 35 and 39 carbon atoms [9,10]. Experimental studies and *ab initio* calculations demonstrated that formulae for determining the chain length by analysis of the LAM in Raman spectra of branched and linear alkanes are the same, because of a minor effect of side alkyl chains on the LAM frequency [9,10,11,12]. On the other hand, the LAM frequency in substituted alkanes like fatty acids, alkylamines, and bromoalkanes is affected by a mass of a terminal group in a hydrocarbon chain [13,14].

However, monitoring of the LAM mode in the spectra of liquid long linear alkanes (*n* ≥ 12) and hydrogenated 1-hexene oligomers may be difficult due to its low intensity and overlapping with D-LAM band [7,15,16]. The D-LAM band corresponds to the superposition of the vibrations, related to the accordion C–C–C motion, like LAM, but in chains having various non-planar conformations [15,16,17]. Recently [7], we found out that the relation of the total number of carbon atoms in the hydrogenated 1-hexene oligomer molecules and the D-LAM peak position (*ν_D-LAM_*) in their Raman spectra is close, although non-identical, to the law suggested by Snyder and Kim for linear alkanes [15,16]:

(1) $$\nu_{D-LAM}\left(cm^{-1}\right)=\frac{5590}{n^{2}}+198$$

The relation between the wavenumber of the symmetric C–C stretching mode, further the C–C mode, and the chain length is more complex [7,18,19,20,21] as compared with that for the LAMs.

Consideration of the Newton’s equation of the motion for a one-dimensional monatomic chain with some additional bonds leads to the following dependence of the C–C mode wavenumber (*ν*_C–C_) on the number (*n*) of carbon atoms in this chain [18]:

(2) $$\nu_{C-C}=\sqrt{\nu_{\infty }^{2}+\beta^{2}{sin}^{2}\frac{\pi }{n}}$$

Here *ν*_∞_ is the wavenumber of this mode for a hypothetical infinite *trans*-chain, β = *s*/π*ac*, where *a* is a repetition period in the alkyl chain, *s* is the velocity of a light wave through the *trans*-chain, and *c* is the velocity of light in a vacuum. This formula excellently describes the relation between the C–C mode wavenumber and the total number of carbon atoms in the molecule for linear alkanes [18,19]. However, experimental studies showed that the C–C mode wavenumber is strongly influenced by “perturbations” in the alkyl chain, such as a double C=C bond in alkenes [20], a heavy end group, or a chain branching [19,21]. As a consequence, breaking of this law is observed for different substances, containing alkyl chains, such as the hydrogenated 1-hexene oligomers [7], alkyl ammonium compounds [19], and *trans*-alkenes [20]. For instance, the observed C–C mode wavenumber of single-chain alkyl ammonium compounds increases with the increasing *n* [19], in clear contradiction with the law (2).

For multi-chain compounds such as solid double-chain alkyl ammonium salts {[CH_3_(CH_2_)_m_]_2_N (CH_3_)_2_}Br with m = 13, 17, the hydrogenated 1-hexene oligomers, the wavenumber, shape, and width of this band are determined by a *trans*-segment length of each chain and by the number of chains [7,21]. Taking into account this fact, the C–C mode could be very attractive for recognizing a chain arrangement in branched alkane dimers, whose backbone chain can adopt both extended and folded conformations [9,22,23].

Interesting experimental observation concerning the polarized Raman spectra of linear alkanes in the region 2800–3200 cm^−1^ was reported by Shemouratov et al. [24], who showed that an intensity ratio of Raman bands, which are assigned to the asymmetric stretching vibrations of CH_2_ and CH_3_ units, monotonically grows with the increasing *n* of linear alkanes. These spectra were recorded at the crossed orientations of the exciting and scattered radiations. One of the aims of the present study was to find similar regularity in the Raman spectra of the hydrogenated α-olefin oligomers.

Thus, Raman spectra of a new practically important class of hydrocarbons—the hydrogenated α-olefin oligomers—contain interesting spectral features that can be used for quantitative structural characterization of these compounds. Detailed analysis of these features based on experimental measurements as well as on the density functional theory (DFT) calculations was a goal of this work.

## 2. Materials and Methods

### 2.1. Sample Preparation

Oligomerization of 1-hexene and 1-octene to prepare individual oligomers was carried out in bulk using 2 mol of the corresponding α-olefin and 10 mmol of bis(η^5^-2,5-dimethyl-4*H*-cyclopenta[*b*]thienyl)dichlorozirconium (IV), activated subsequently by 200 mmol of TIBA and 100 mmol of modified methylalumoxane MMAO-12, as reported earlier [1]. After treatment with a minimal quantity of ethanol and water, the reaction mixtures were distilled in vacuo collecting the fractions up to 280 °C/0.2 Torr. The isomeric purity of the products was confirmed by gas chromatography, and by the absence of 4.9–6.0 ppm peaks in ^1^H NMR spectra [1]. The combined fractions of each experiment were hydrogenated in the presence of Pd/Al_2_O_3_ catalyst (0.1% Pd, 0.02% by mol) at the temperature of 90 °C and hydrogen pressure of 10 Bar within 8 h. Each sample was subjected by fractional distillation to obtain hydrogenated oligomers. Boiling points (T, °C/pressure, Torr) were 80/7 (H2H), 120/0.5 (H3H), 145/0.2 (H4H), 190/0.2 (H5H), 105/0.8 (O2H), 166/0.8 (O3H), 212/0.8 (O4H), 242/0.2 (O5H).

### 2.2. Raman Measurements

In order to record Raman spectra of the hydrogenated 1-octene and 1-hexene oligomers, we used two Raman setups. The first setup, an inVia Raman microscope (Renishaw, Great Britain), equipped with CCD camera and 785 nm laser source, allowed the spectra to be recorded with the spectral resolution better than 1.5 cm^−1^. The scattered light was collected in the backscattering geometry using a 50× lens (N.A. 0.75) and exposure time of 10 s. The sample drops were placed on aluminum plate-holder, no cover glass was used. The laser power on a sample was 2.4 mW. This experimental setup was used to record spectra only in the region 1000–1600 cm^−1^, with the exception of the isomers O3H and H4H. We did not carry out measurements in the region 50–600 cm^−1^, because of strong Rayleigh scattering, and in the region 2700–3200 cm^−1^, due to the low sensitivity of the CCD camera.

The second Raman setup included Sapphire SF 532 laser (Coherent Inc., Santa Clara, CA, USA) with the 532.0 nm excitation line, double monochromator (U1000, Jobin Yvon, France), and a photomultiplier detector. To record polarized spectra, the analyzer was placed in front of the monochromator entrance slit. The spectra were recorded in a 90°-scattering geometry with the spectral resolution of 5 cm^−1^. Using the second Raman setup, we recorded non-polarized and polarized Raman spectra of the hydrogenated oligomers and liquid linear alkanes C_n_H_2n+2_ with *n* = 5–15 in the regions 50–600 and 2700–3200 cm^−1^. We used Raman spectra of CCl_4_ for wavenumber calibration for the spectra recorded by different experimental setups.

We did not detect any change in the Raman bands at different exposure times of the spectra. For this reason, we concluded that possible temperature-induced structural changes because of local heating of the samples by the laser radiation are negligible. 

### 2.3. Quantum-Chemical Calculations

We applied the density functional theory (DFT) for computational modeling, which included: an optimization of molecule geometry and calculation of the total energy for a number of conformations, calculations of the wavenumbers, Raman scattering activities (RSAs), and depolarization ratios for all the optimized molecule conformations.

The features of the DFT calculations of molecular geometry and Raman spectra, including justification of the choice of the functional and basis, were described in detail in our recent DFT study of Raman spectra of linear alkanes (pentane, hexane, and octadecane) [25], presented in the Supporting Information (SI). The calculations employed the OLYP functional [26], the Gaussian orbitals 4z basis (Table S1 in the SI), and the PRIRODA software (M.V. Lomonosov Moscow State University, Russian Federation) for quantum-chemical calculations [27,28,29]. We carried out the calculations for an isolate molecule using the harmonic oscillator approximation.

The DFT calculated wavenumbers for the C–C mode and the LAM of linear alkanes were on average 3 and 12 cm^−1^ lower than the respective observed wavenumbers. Table A1 in the Appendix A shows the observed and calculated wavenumbers of these modes for linear alkanes with *n* = 5–18, 20. The DFT calculated wavenumbers in Table A1 (Appendix A) belong to the vibrations of the molecules in the *all trans* conformation.

## 3. Results and Discussion

### 3.1. Spectral Region 60–500 cm^−1^

**Figure 2:**
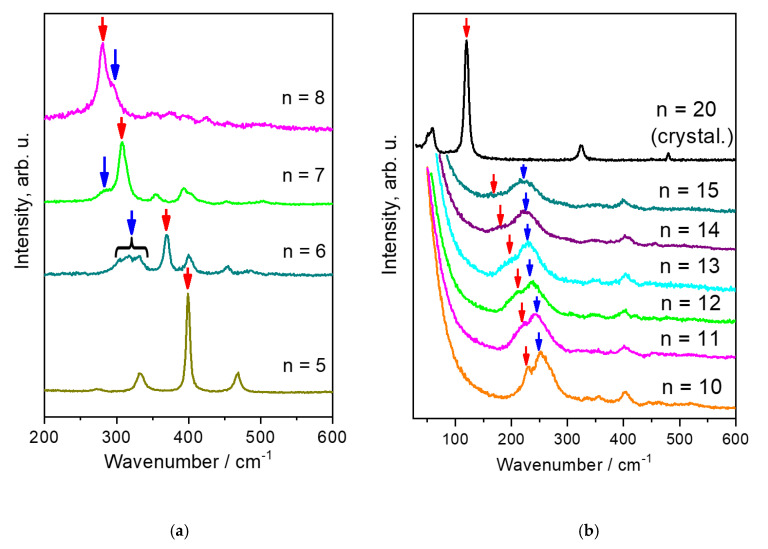
Raman spectra of linear short-chain (**a**) and long-chain (**b**) linear alkanes in the region of longitudinal acoustic mode (LAM; red arrows) and disorder LAM (D-LAM; blue arrows) bands.

**Figure 3:**
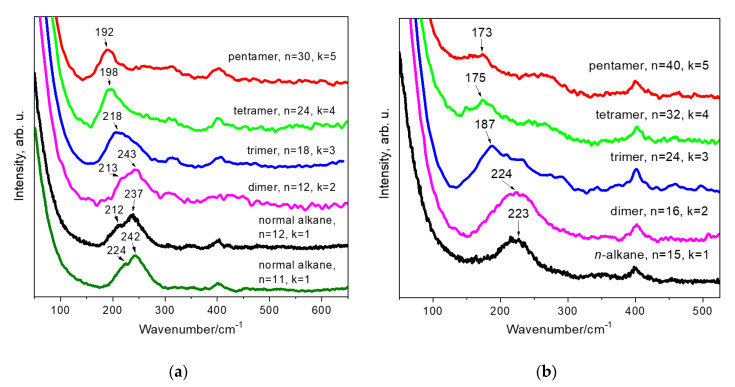
Raman spectra of the hydrogenated 1-hexene (**a**) and 1-octene (**b**) oligomers, as well as Raman spectra of linear alkanes C_11_H_24_, C_12_H_26_ (**a**) and C_15_H_32_ (**b**) in the region of the LAM and D-LAM.

Figure 2 shows Raman spectra of the short-chain and long-chain linear alkanes in the region of the acoustic modes. In Figure 3, the Raman spectra of linear alkanes with *n* = 11, 12, and 15 are compared with the spectra of the hydrogenated 1-hexene and 1-octene oligomers.

To interpret features of the Raman spectra of the hydrogenated α-olefin oligomers, let us first consider the Raman spectra of linear alkanes. At room temperature the Raman spectra (Figure 2a) of short-chain alkanes (*n* < 9) demonstrate two very prominent bands. The high-intense narrow bands belong to the LAM, which is the C–C–C bend vibrations in extended molecules in the *all trans* conformation. The low-intense broad bands are assigned to the D-LAM [15]. With an increasing length of alkyl chain until *n* = 16, an amount of molecules in non-planar conformations in liquid alkanes grows, while the relative content of molecules in the *all trans* conformation decreases. It results in diminishing the LAM band intensity, while the intensity of the D-LAM band, on the contrary, gives rise (Figure 2). Consequently, only the D-LAM band is evident in the Raman spectra of liquid long-chain linear alkanes (Figure 2b). However, this band vanishes in Raman spectra of solid linear alkanes, which contain only extended chains in the *all trans* conformation. Because of this, the high-intense narrow band related to the LAM reappears in the spectra of solid alkanes (Figure 2b).

Table 1 shows the peak positions of the LAM and D-LAM bands as well as the symmetric C–C stretching mode for the hydrogenated oligomers of α-olefins and the selected linear alkanes (with *n* = 6, 8, 12, 15, 18).

If we consider the vibrations of chains in the oligomer molecules as independent and apply directly our knowledge about Raman spectra of linear alkanes, we should expect an appearance of Raman bands about 317–370 cm^−1^ for the 1-hexene oligomers and about 280–296 cm^−1^ for the 1-octene oligomers (i.e., with wavenumbers), related to the D-LAM and LAM in alkyl chains with 5-8 carbon atoms (Table 1 and Table A1). Thus, if this approach is correct, the Raman spectra of the oligomers in the low-wavenumber region should be similar to that of liquid short-chain alkanes (*n* = 5–8). In addition, no shift of the LAM and D-LAM bands with increasing the number of carbon atoms is expected.

**Figure 4:**
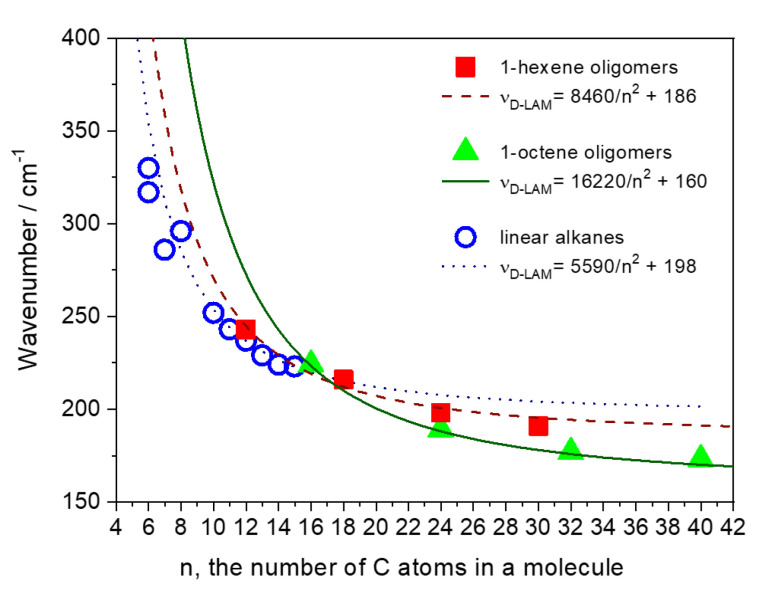
Peak position of the D-LAM band versus the total number (*n*) of C atoms for liquid linear alkanes and the hydrogenated 1-hexene and 1-octene oligomers. Symbols are the observed D-LAM wavenumbers: 1-hexene (■ and 1-octene (▲) oligomers, linear alkanes (○), curves (solid, dash and dot) are the fitting functions $\nu_{D\text{-}\mathrm{LAM}}\left({\mathrm{cm}}^{-1}\right)=A/n^{2}+b$ (see text). The measurement errors of the peak positions are less than 3 cm^−1^.

However, in the low-wavenumber area the Raman spectra of the hydrogenated α-olefin oligomers, like that of the liquid long-chain alkanes (*n* = 10–15), demonstrate a broad band (Figure 2b and Figure 3). This band, which we assign to the D-LAM, shifts to lower wavenumbers when the total number of carbon atoms increases in an oligomer molecule. Figure 4 shows the dependences of the peak position of the D-LAM band on the total number of C atoms for liquid linear alkanes and the oligomers under study. We found out that *ν_D-LAM_* in the Raman spectra of the hydrogenated oligomers, like that of the D-LAM band for linear alkanes, depends on *n* as:

(3) $$\nu_{D-LAM}\left(cm^{-1}\right)=\frac{A}{n^{2}}+b$$

where *A* and *b* are the constants. Note that the coefficients *A* and *b* are different for the 1-hexene (*A* = 8460 cm^−1^, *b* = 186 cm^−1^) and 1-octene (*A* = 16220 cm^−1^, *b* = 160 cm^−1^) oligomers and also for linear alkanes (*A* = 5580 cm^−1^, *b* = 198 cm^−1^), although the relation (1), derived for linear alkanes, is still correct for the dimer and trimer of 1-hexene, and for the 1-octene dimer (Figure 4). The measurement error at determination of the coefficients *A* and *b* does not exceed 5%.

We observed the noticeable discrepancy in the D-LAM wavenumbers for the oligomers in comparison with the wavenumbers, which the relation (1) predicts for linear alkanes, that appeared for *n* ≥ 24 (i.e., for 1-octene trimer), as well as tetramers and pentamers of both 1-hexene and 1-octene. This discrepancy became more noticeable with the increasing length of branched chains in the oligomer molecule.

It is important that the relation between the D-LAM wavenumber and *n*, valid for linear alkanes, is losing for the oligomers with *n* ≥ 24, and the D-LAM wavenumber depends on both the total number of carbon atoms in the molecule and the length of side chains. For instance, the D-LAM wavenumbers are 198 and 187 cm^−1^ for the isomers H4H and O3H, respectively (Figure 4, Table 1). Both these compounds have the same chemical formula C_24_H_50_. Thus, first, the conformational composition of molecules in the hydrogenated α-olefin oligomers depends on the total number of carbon atoms and a chain length and the number of monomer (1-hexene or 1-octene) subunits. Second, the D-LAM band can be used for determination of a total number of carbon atoms in a molecule of these oligomers, if length of the monomer chain is known. Note, the number of possible conformations grows with increasing *n*. However, the probabilities of existence of various conformations differ significantly and, as a consequence, their contributions in the D-LAM bands are different. The “narrow” D-LAM bands in the spectra of the tetramers and pentamers can be explained by the presence of favorable conformations, whose probabilities of existence exceed significantly that for the other conformations. Along with the narrow D-LAM bands, we observed the low-intensity scattering about 250–350 cm^−1^ in the spectra of the tetramers and pentamers. We consider that this scattering also belongs to the D-LAM bands and originates from numerous conformations with low probabilities of existence. 

### 3.2. Symmetric C–C Stretching Mode

#### 3.2.1. Spectral Studies

**Figure 5:**
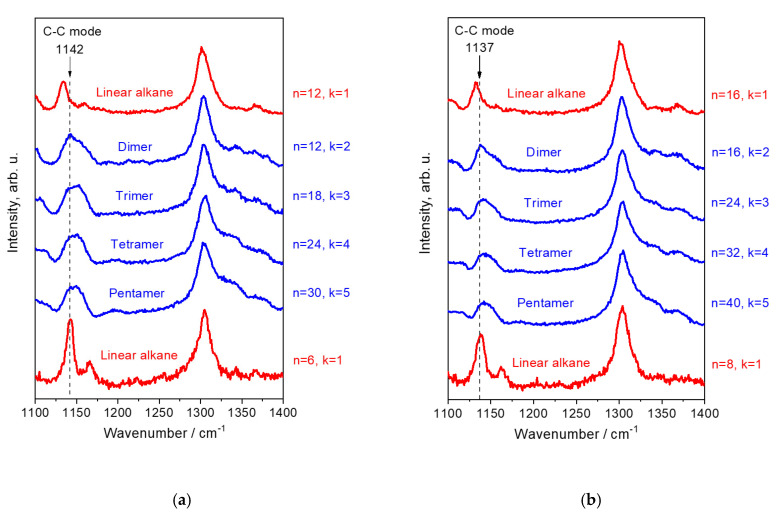
Raman spectra of the hydrogenated 1-hexene (**a**) and 1-octene (**b**) oligomers and linear alkanes with *n* = 6, 12 (**a**) and *n* = 8, 16 (**b**) in the region of the symmetric C–C stretching mode.

Figure 5 shows the Raman spectra of the hydrogenated 1-hexene and 1-octene oligomers in the region 1110–1400 cm^−1^. For comparison, this figure displays the Raman spectra of linear alkanes with *n* = 6, 8, 12, and 16. The Raman band, which belongs to the C–C mode (the symmetric C–C stretching mode), is localized in the spectral region 1100–1200 cm^−1^.

We defined the spectral characteristics (the wavenumbers and the full widths at the half-maximum (FWHM)) of the C–C band in Table 1. The wavenumbers and FWHMs for the dimers were determined by the spectrum deconvolution using two lines and nonlinear (second-order polynomial) background. The profile of each line was described as a weighted sum of Gaussian and Lorentzian functions [25]. For other oligomers, the wavenumber values, obtained using the spectrum deconvolution, demonstrated a poor accuracy (about 5 cm^−1^). For this reason, the wavenumbers given in Table 1 of the C–C mode for trimers, tetramers, and pentamers were evaluated at intensity maximums of their Raman bands.

**Figure 6:**
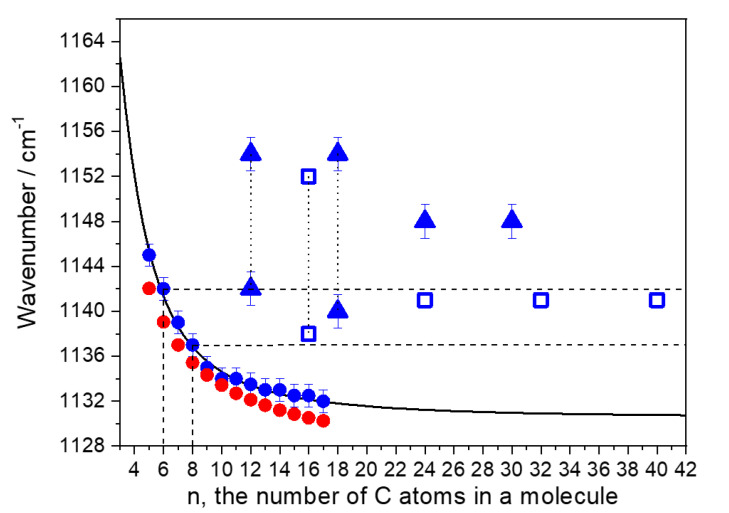
Peak position of the C–C mode for the hydrogenated 1-hexene (▲) and 1-octene (□) oligomers as well as for linear alkanes (●,●) versus the number of carbon atoms in a molecule. Symbols ▲,□,● are the observed wavenumbers, symbols ● are the DFT wavenumbers, the solid curve is the theoretical law for linear alkanes at *ν*_∞_ = 1130.5 cm^−1^ and β = 313 cm^−1^ (see text: Equation (2)). The vertical dotted lines drawn through triangle or square symbols connect the data attributed to the particular substance of the H2H, H3H, or O2H compounds (see Table 1 and Figure 5). The measurement errors of the peak positions are also indicated.

Figure 6 depicts the observed wavenumbers of the C–C mode for linear alkanes and the oligomers under study versus the number of carbon atoms in a molecule, as well as the function (2) and the DFT wavenumbers of the C–C mode for the *all trans* alkyl chains. We take the coefficients *ν*_∞_ = 1130.5 cm^−1^ and β = 313 cm^−1^ in the expression (2) from the publication [19].

The Raman spectra of the oligomers and linear alkanes in the region 1110–1400 cm^−1^ differ dramatically, in the first turn, in the spectral features of the C–C bands (Figure 5).

In the Raman spectra of liquid linear alkanes, the C–C mode related to the vibrations in the *trans*-sequences [17,19,25,31,32] demonstrates narrow and symmetric band with FWHM = 7–13 cm^−1^ (Table 1, Figure 5). The observed wavenumbers for the band follow the theoretical law (2) at *ν*_∞_ = 1130.5 cm^−1^ and β = 313 cm^−1^ (Figure 6). Additionally, they correlate perfectly with the DFT wavenumbers calculated for the *all trans* alkyl chains of various length (Figure 6). Taking into account such excellent agreement in the observed and DFT wavenumbers, we concluded that for linear alkanes a contribution of other, different from the *all trans* conformations in the intensity of the C–C band, is negligible. 

The C–C mode in the oligomer Raman spectra manifests as a broad and, for most of the samples, asymmetric band. The C–C bands of the 1-hexene and 1-octene dimers demonstrate the peak intensity at 1142 and 1138 cm^−1^, respectively. These wavenumbers correspond to the *trans*-segments, consisting of six and eight carbon atoms for the dimers of 1-hexene and 1-octene, respectevely, but not to *trans*-segments of 11–12 and 15–16 carbon atoms, as might be expected from analysis of the spectra of their isomers—linear alkanes C_12_H_26_ and C_16_H_34_ (Figure 5 and Figure 6). In contrast with the regularities for linear alkanes, the C–C band of the oligomers shifts to lower wavenumers and changes its shape with an increase in the number of the branched chains (Table 1, Figure 5 and Figure 6). We explain such behavior of the C–C band as well as its asymmetric bandshape and large FWHM (~30 cm^−1^) by presence of additional scattering from the shorter *trans* segments, containing no more than five and seven carbon atoms for the 1-hexene and 1-octene oligomers, respectevely.

**Figure 7:**
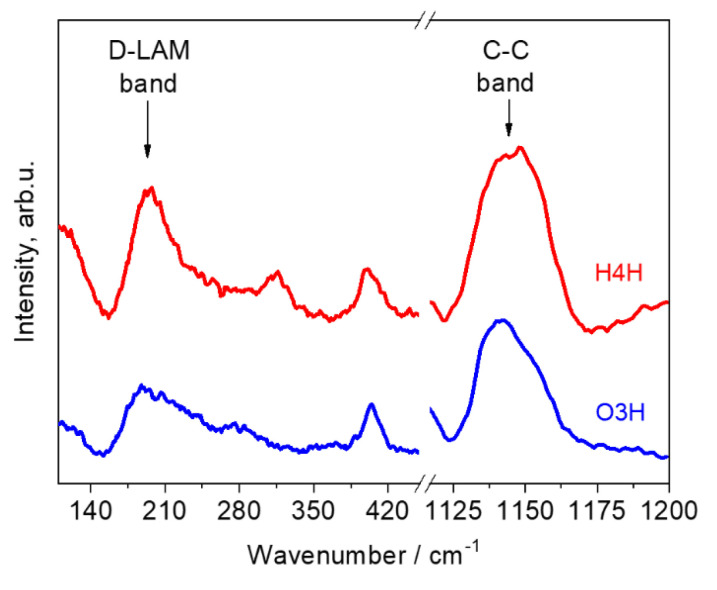
D-LAM and C–C bands of isomers with formula C_24_H_50_: 1-hexene tetramer (H4H) and 1-octene trimer (O3H).

Therefore, the peak position of the C–C mode can be used as a measure of length of the branched chains, but not as a characteristic of the total number of C atoms in the oligomer molecules. Thus, the jont monitoring of the D-LAM and the C–C mode allows for distinguishing the hydrocarbon isomers—linear alkanes and the 1-hexene and 1-octene oligomers. For instance, Figure 7 shows the D-LAM and C–C bands of the isomers under study with chemical formula C_24_H_50_: 1-hexene tetramer (H4H) and 1-octene trimer (O3H). As can be seen from this figure, the spectra of these oligomers demonstrate differences in the peak positions and shapes of the D-LAM and C–C bands.

#### 3.2.2. DFT Calculations

The molecular architecture of the trimers, tetramers, and pentamers implies an existence of side chains, arranged at some angles relative to each other. However, the double chain structure is not obvious for the dimers. Moreover, as mentioned in the introduction, molecules of some CH_3_-cental branched alkanes adopt the extended-chain conformation in solids [9,10].

To clarify this question, and to support our experimental study, we applied the DFT calculations of the relations of the C–C mode spectral characteristics and the conformational arrangement of molecules in the 1-hexene and 1-octene dimers.

**Figure 8:**
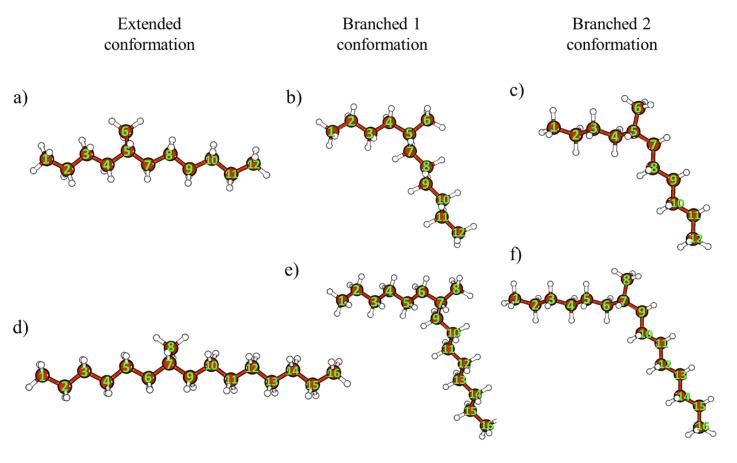
Density functional theory (DFT) calculated conformations of dimers of 1-hexene (**a**–**c**) and 1-octene (**d**–**f**): extended-chain conformation (**a**,**d**), branched 1 chain conformation (**b**,**e**), branched 2 chain conformation (**c**,**f**).

Figure 8 depicts the DFT calculated conformations for the 1-hexene and 1-octene dimers: the conformation of extended chain and two branched conformations. The total energies of molecules in these conformations differ slightly (about 0.15 kcal/mol) from each other. The geometry parameters (the C–C bond lengths, bond angles, and torsional angles), total energies (ΔE) for the ground state (or electron level), and total energies corrected for zero point vibration energies (ZPVE) ΔE_0_, as well as the wavenumbers *ν*_C–C_ and RSA of the C–C modes, are reported in Table 2 and Table 3 for the 1-hexene and 1-octene dimers, respectively. The negative values of the torsion angles correspond to a clockwise rotation of the last carbon bond.

**Figure 9:**
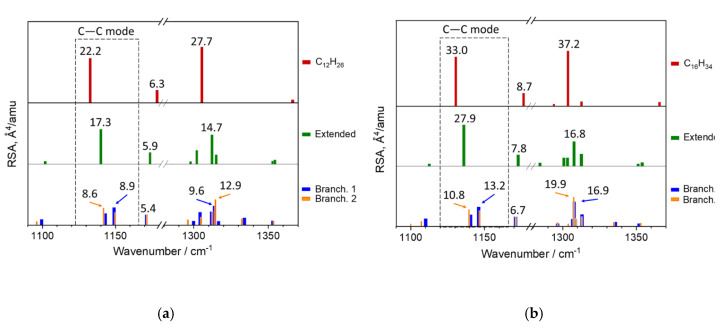
DFT calculated Raman spectra of molecules in different conformations (the extended, branched 1 and 2 chain conformations): linear alkane C_12_H_26_ (**a**) and H2H (**a**), linear alkane C_16_H_34_ and O2H (**b**) in the region 1100–1360 cm^−1^.

Figure 9 illustrates the DFT calculated Raman spectra of molecules in these three conformations in the region 1100–1350 cm^−1^. For comparison, this figure also demonstrates the DFT Raman spectra of extended-chain conformations for linear alkanes C_12_H_26_ and C_16_H_34_. In addition, we list the atomic coordinates, the absolute values of total energy, and the DFT calculated Raman spectra of the dimer conformations in Tables S2–S4 in the SI.

The extended-chain conformation of the oligomer dimers is similar to the *trans* zigzag conformation of linear alkanes and possesses the lowest value of the potential energy in comparison with the other molecular geometries, analyzed in this study (Figure 8, Table 2 and Table 3). This conformation was obtained as the result of the geometry optimization started from the initially chosen *all trans* chain conformation. We took ΔE and ΔE_0_ (ZPVE corrected ΔE) values for the extended chain conformation as zero. The values ΔE and ΔE_0_ in Table 2 and Table 3 are in excess of the corresponding energies about the ones for the extended-chain conformations. The absolute values of total energy and ZPVE are reported in SI (Table S4).

The both branched conformations consist of two planar chains, which connect with each other by a *trans-gauche-gauche-trans* segment (Figure 8, Table 2 and Table 3). These conformations differ in a position of the central CH_3_-group. The C atom of the CH_3_ group is included in a *trans*-conformer with the shorter alkyl chain in the branched 1 chain conformation. In case of the branched 2 conformation, the *trans*-conformer is formed with the longer alkyl chain (Figure 8). The torsion angles C3–C4–C5–C6 and C8–C7–C9–C10 for 1-hexene dimer are presented in Table 2, the torsion angles C5–C6–C7–C8 and C6–C5–C7–C8 for 1-octene dimer are given in Table 3.

DFT calculations confirmed potential of analysis of the symmetric C–C stretching mode to distinguish the extended and branched chain conformations. According to the DFT calculations, Raman spectra of the dimers (or alkanes) in the extended-chain conformation contains only one Raman-active line, which corresponds to the symmetric C–C stretching mode (Figure 9). By contrast, two lines, related to the symmetric C–C stretching mode, appear in the case of the branched conformations. DFT wavenumbers for the branched conformations are shifted towards higher wavenumbers as compared with the DFT wavenumbers for the extended-chain conformation. Sums of the RSA values of these modes are close to the RSA for the extended-chain conformation (Figure 9, Table 2 and Table 3).

For example, for the 1-hexene dimers:

- the extended-chain conformation: RSA_1139_ = 17.27
- the branched 1 chain conformation: RSA_1143_ + RSA_1149_ = 14.59
- the branched 2 chain conformation: RSA_1142_ + RSA_1150_ = 14.98.

For the 1-octene dimers:

- the extended-chain conformation: RSA_1136_ = 27.87
- the branched 1 chain conformation: RSA_1140_ + RSA_1146_ = 21.40
- the branched 2 chain conformation: RSA_1140_ + RSA_1147_ = 22.40.

Here, RSA’s subscript means the DFT wavenumber of the vibration, which this RSA value corresponds to.

Let us analyze in detail the doublet of the lines, related to the C–C mode of the dimers in the branched conformations. 

The first component, the in-phase symmetric C–C stretching mode with the DFT wavenumber about 1142 and 1140 cm^−1^ for the 1-hexene and 1-octene dimers, respectively, originates from a movement, in which C–C bonds in both alkyl chains expand and contract simultaneously. The second component, the out-phase symmetric C–C stretching mode, with the DFT wavenumber about 1150 and 1146 cm^−1^ for the 1-hexene and 1-octene dimers, respectively, belongs to a movement in which C–C bonds in one alkyl chain expands, whereas the C–C bonds in another chain shrinks. We support our conclusions by animation of the vibrations for the C–C mode for the H2H molecule (see Video Supporting Information in-phase. mp4 and out-phase. mp4).

A comparison of the experimental and DFT calculated spectra revealed the paradoxical, at first glance, result. The extended-chain conformation is the energetically most favorable state for the dimers (H2H or O2H), and the RSA values of the symmetric C–C stretching mode for this conformation are high enough for manifestation of the corresponding line in the experimental spectra (Figure 9). Nevertheless, we could not identify the lines, related to the extended-chain conformation, in the dimer spectra by monitoring the symmetric C–C stretching mode. By contrast, the peak positions and intensities of the calculated lines of the branched conformations, having the higher energies E and E_0_, correlates excellently with that of the lines, observed in the experimental spectra. This unexpected result can be explained by the assumption that the Raman bands of the branched-chain conformations mask the contribution from the extended-chain conformation.

By comparing the DFT calculated Raman spectra, we concluded that there is no possibility to distinguish the branched 1 and 2 chain conformations by monitoring the symmetric C–C stretching mode (Figure 9, Table 2 and Table 3). Therefore, for analysis of the experimental spectra in this spectral region these two conformations should be considered as one generalized branched-chain conformation, with the RSA values of the in-phase and out-phase symmetric C–C stretching modes equal to the sums of the corresponding values for the branched 1 and 2 chain conformations.

Taking into account the negligible difference in the calculated energies of the branched 1 and 2 chain conformations, we concluded that the relative contents of the molecules in these conformations should be comparable. In this case, the total content of the molecules in the branched 1 and 2 chain conformations can even exceed the content of the molecules in the extended-chain conformation. Thus, the generalized branched chain conformation of molecules of the liquid 1-hexene and 1-octene dimers are more probable than the extended chain conformation.

### 3.3. The Region 2750–3150 cm^−1^

**Figure 10:**
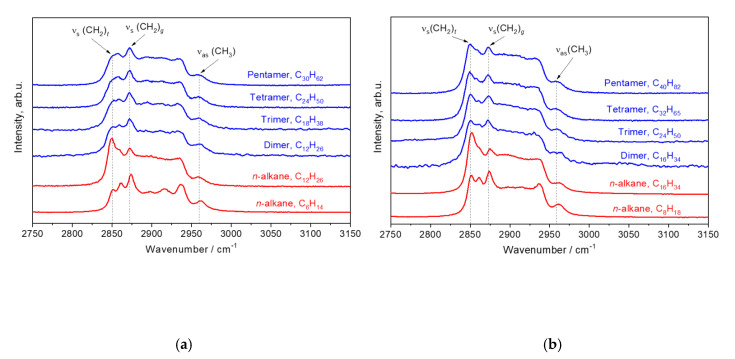
Non-polarized Raman spectra of the hydrogenated 1-hexene (**a**) and 1-octene (**b**) oligomers and linear alkanes C_6_H_14_, C_8_H_18_, C_12_H_26_, and C_16_H_34_ in the region 2750–3150 cm^−1^.

Figure 10 depicts non-polarized Raman spectra of the hydrogenated 1-hexene and 1-octene oligomers and the linear alkanes C_6_H_14_, C_8_H_18_, C_12_H_26_, and C_16_H_34_ in the region 2750–3150 cm^−1^. By using the results of the previous work on linear alkanes [24], we assigned the bands *ν*_s_(CH_2_)*_t_* at 2850 cm^−1^ and *ν*_s_(CH_2_)*_g_* at 2874 cm^−1^ in the non-polarized oligomer spectra to the symmetric stretching vibrations of CH_2_ units in *trans* and *gauche* conformers, respectively (Figure 10). The band *ν*_as_(CH_3_) at 2960 cm^−1^ is related to the asymmetric stretching vibrations of CH_3_ units [31,32]. These spectra of the hydrogenated α-olefin oligomers do not show noticeable alterations with a change of the length or number of branched chains. For this reason, we concluded that the non-polarized spectra in the region 2750–3150 cm^−1^ provide little information on the oligomer structure, and the non-polarized spectra in this region are not analyzed in this work in detail.

**Figure 11:**
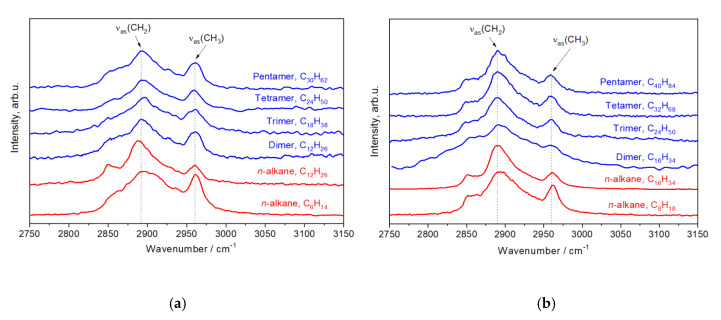
Polarized Raman spectra of the hydrogenated 1-hexene (**a**) and 1-octene (**b**) oligomers and linear alkanes C_6_H_14_, C_8_H_18_, C_12_H_26_, and C_16_H_34_ in the region 2750–3150 cm^−1^. These spectra are recorded at the crossed orientation of the exciting and scattered radiation.

The polarized spectra, which are recorded at the crossed orientation of the exciting and scattered radiations in the region 2750–3150 cm^−1^, are more attractive for structural analysis of the α-olefin oligomers. Figure 11 shows such Raman spectra of the hydrogenated 1-hexene and 1-octene oligomers. In addition, we display in Figure 11 the polarized Raman spectra of the linear alkanes C_6_H_14_, C_8_H_18_, C_12_H_26_, and C_16_H_34_ for comparison. According to our previous Raman studies on linear alkanes (with *n* = 5–17) [24], the most prominent bands in the polarized Raman spectra, which are recorded at the crossed orientation of the exciting and scattered radiations, belong to the asymmetric stretching vibrations of CH_2_ and CH_3_ units. By comparing the polarized Raman spectra of the oligomers and alkanes, we assigned the bands *ν*_as_(CH_2_) at ~2890 cm^−1^ and *ν*_as_(CH_3_) at ~2960 cm^−1^ in the spectra of the oligomers to the asymmetric stretching vibrations of CH_2_ and CH_3_ units, respectively (Figure 11).

Table 4 reports the wavenumbers of *ν*_as_(CH_2_) and *ν*_as_(CH_3_) for the hydrogenated 1-hexene and 1-octene oligomers and linear alkanes C_6_H_14_, C_8_H_18_, C_12_H_26_, and C_16_H_34_. Note that these wavenumbers were determined using the peak intensity of the corresponding band (i.e., without any spectral deconvolition). We found out that the position of the *ν*_as_(CH_2_) band shifts from ~2895 to 2890 cm^−1^ with an increase in the length of the monomer subunit, but it does not depend on the total number of C atoms in the oligomer molecules. Thus, the peak position of *ν*_as_(CH_2_) band can be an additional marker for determining the branched chain length, and, in this way, to distinguish the 1-hexene oligomers and 1-octene oligomers.

The most interesting result of the analysis of the polarized Raman spectra in this region concerns the relation of the ratio of the peak intensities $I_{CH_{3}}/I_{CH_{2}}$ of the bands *ν*_as_(CH_3_) and *ν*_as_(CH_2_) and the relative content $n_{CH_{3}}/n_{CH_{2}}$ of CH_3_ and CH_2_ units.

**Figure 12:**
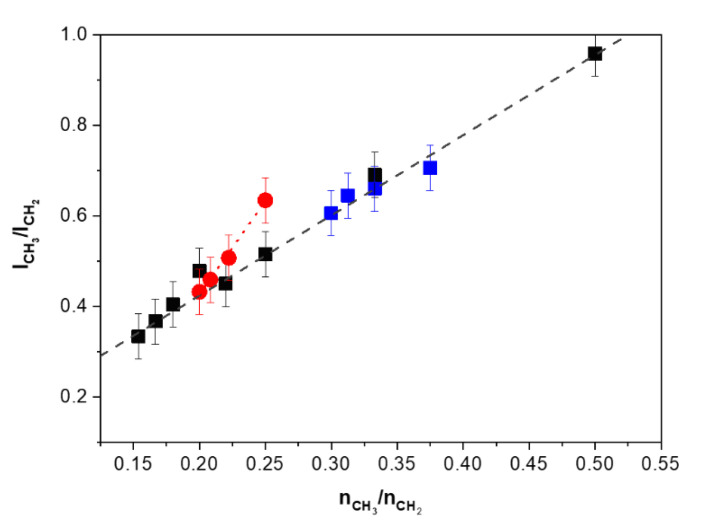
The ratio $I_{CH_{3}}/I_{CH_{2}}$ *versus* the relative content $n_{CH_{3}}/n_{CH_{2}}$ of CH_3_ and CH_2_ units for the hydrogenated 1-hexene oligomers (■), 1-octene oligomers (●), and linear alkanes (■). Symbols ■, ●, ■ are the experimental data; dark grey and red dash lines are the linear functions (4) and (5), respectively. The measurement errors of the intensity ratio are also indicated.

Figure 12 shows the ratio of the peak intensities $I_{CH_{3}}/I_{CH_{2}}$ of the bands *ν*_as_(CH_3_) and *ν*_as_(CH_2_) as functions of the relative content $n_{CH_{3}}/n_{CH_{2}}$ of CH_3_ and CH_2_ units for the hydrogenated 1-hexene and 1-octene oligomers as well as for linear alkanes. 

We found out that the observed values of the ratio $I_{CH_{3}}/I_{CH_{2}}$ are excellently described by the following linear functions:

For both the hydrogenated 1-hexene oligomers and linear alkanes:

(4) $$\frac{I_{CH_{3}}}{I_{CH_{2}}}=1.77\times \frac{n_{CH_{3}}}{n_{CH_{2}}}+0.07$$

For the hydrogenated 1-octene oligomers:

(5) $$\frac{I_{CH_{3}}}{I_{CH_{2}}}=4.07\times \frac{n_{CH_{3}}}{n_{CH_{2}}}-0.39$$

For the relations 4 and 5, we used the peak intensities of the bands without spectral deconvolution. However, the spectra in the region 2750–3150 cm^−1^ are very complex, because of the overlapping of numerous bands. The other vibrations, in particular, the overtones of the deformation vibrations of CH_2_ groups, contribute to the intensity at the wavenumbers of the asymmetric stretching vibrations of CH_2_ and CH_3_ units [24]. This can be the reason that the oligomers of 1-octene exhibit a relation between $I_{CH_{3}}/I_{CH_{2}}$ and $n_{CH_{3}}/n_{CH_{2}}$, which is different from that for the 1-hexene oligomers and linear alkanes. Nevertheless, the analysis of the ratio of the peak intensities $I_{CH_{3}}/I_{CH_{2}}$ of the bands *ν*_as_(CH_3_) and *ν*_as_(CH_2_) allows determination of the relative content $n_{CH_{3}}/n_{CH_{2}}$ of CH_3_ and CH_2_ units. This ratio can serve also as a marker of the chain amount, if the branched chain length is known.

## 4. Conclusions

In the course of the work we carried out experimental studies and DFT calculations of Raman spectra of a novel family of liquid branched alkanes (with a general chemical formula C_n_H_2n+2_)—the hydrogenated α-olefin oligomers such as dimers, trimers, tetramers, and pentamers of 1-hexene and 1-octene.

We found out that the joint monitoring of the D-LAM, the symmetric C–C stretching mode, as well as the asymmetric stretching vibrations of CH_2_ and CH_3_ groups allows for characterization of these oligomers in terms of the number and length of CH_2_-chains and to distinguish these compounds from linear alkanes.

If the D-LAM wavenumber *ν_D-LAM_* is known, the total number *n* of carbon atoms in a molecule of these oligomers can be determined by the simple relations:

$$n^{2}=\frac{8460}{\left(\nu_{D-LAM}-186\right)}\;\mathrm{for}\;\mathrm{the}\;1\text{-}\mathrm{hexene}\;\mathrm{oligomers};$$

$$n^{2}=\frac{16600}{\left(\nu_{D-LAM}-160\right)}\;\mathrm{for}\;\mathrm{the}\;1\text{-}\mathrm{octene}\;\mathrm{oligomers}.$$

The peak position and the shape of the symmetric C–C stretching band are sensitive to both the number and length of branched CH_2_-chains. The ratio of the contents of CH_2_ and CH_3_ groups in these branched hydrocarbons can be determined by using intensities of the bands, related to the asymmetric stretching vibrations of CH_2_ and CH_3_ groups, in the polarized spectra, which should be recorded at the crossed orientation of the exciting and scattered radiations.

The DFT calculated Raman spectra for different conformations of the oligomer dimers confirmed the splitting of the symmetric C–C stretching mode on two components (in-phase and out-phase vibrations) for the case of co-existence of two different-length arms with non-parallel arrangement. The branched chain conformation of molecules of the 1-hexene and 1-octene dimers really is the combination of two similar conformations (Figure 8). It is more favorable than the extended-chain conformation for the liquid hydrogenated 1-hexene and 1-octenene dimers.

In view of the fact that Raman spectroscopy was used recently [33] in the investigation of the rheology of poly-α-olefin oils, the results of our study can be usefully applied in the further application of this technique in the analysis and development of the modern PAOs.

## Figures and Tables

**Table 1:** The observed wavenumbers of some Raman-active bands of the hydrogenated 1-hexene and 1-octene oligomers and linear alkanes with *n* = 6, 8, 12, 16, and 18 at room temperature.

Sample	D-LAM	LAM	C–C Mode	
	Wavenumber, cm^−1^	Wavenumber, cm^−1^	Wavenumber, cm^−1^	FWHM ^1^, cm^−1^
1-hexene oligomers				
H2H, C_12_H_26_, *n* = 12, k = 2 ^2^	243	Lines are not observed at room temperature	1142, 1154 (sh) ^3^	16, 21
H3H, C_18_H_38_, *n* = 18, k = 3	218		1140 (sh)/1154	27
H4H, C_24_H_50_, *n* = 24, k = 4	198		1148	30
H5H, C_30_H_62_, *n* = 30, k = 5	192		1148	30
1-octene oligomers				
O2H, C_16_H_34_, *n* = 16, k = 2	224	Lines are not observed at room temperature	1138, 1152	12, 21
O3H, C_24_H_50_, *n* = 24, k = 3	187		1142	33
O4H, C_32_H_66_, *n* = 32, k = 4	175		1141	33
O5H, C_40_H_82_, *n* = 40, k = 5	173		1142	33
Linear alkanes (k = 1)				
C_6_H_14_, *n* = 6	317/330	370	1142	10
C_8_H_18_, *n* = 8	296	280	1137	13
C_11_H_24_, *n* = 11	243	221	1134	11
C_12_H_26_, *n* = 12	237	212	1134	11
C_15_H_32_, *n* = 15	223	Lines are not observed at room temperature	1133	11
C_18_H_38_, *n* = 18	Absent at room temperature	133 ^4^	1130	7

^1^ FWHM is the full width at the half-maximum of the Raman band. ^2^ k is the number of chains in a molecule. ^3^ sh = shoulder. ^4^ Parker S.F. et al. [30].

**Table 2:** DFT calculated characteristics of the hydrogenated 1-hexene dimer conformations.

Characteristics		Conformations		
		Extended	Branched 1	Branched 2
ΔE, kcal/mol		0	0.112	0.112
ΔE_0_, kcal/mol		0	0.144	0.145
C–C bond length, Å		1.54	1.54	1.54
Torsion angle (terminal), °	C1–C2–C3–C4	−180.0	−179.3	179.7
Torsion angles (central), ° *trans−gauche−gauche−trans* part	C2–C3–C4–C5	−175.3	178.7	−177.3
	C3–C4–C5–C7	168.3	72.8	165.3
	C3–C4–C5–C6	−65.8	−161.5	−70.0
	C6–C5–C7–C8	65.9	70.5	161.6
	C4–C5–C7–C8	−168.2	−164.8	−72.7
	C5–C7–C8–C9	175.6	177.8	−178.7
Torsion angle (terminal), °	C7–C8–C9–C10, C8–C9–C10–C11, C9–C10–C11–C12	59.9	−59.9	179.8
*ν*_C-C_, cm^−1^ (RSA, Å^4^/amu)		1139 (17.265)	1143 (5.87)	1142 (8.64)
			1149 (8.87)	1150 (6.34)

**Table 3:** DFT calculated characteristics of the hydrogenated 1-octene dimer conformations.

Characteristics		Conformations		
		Extended	Branched 1	Branched 2
ΔE, kcal/mol		0	0.108	0.107
ΔE_0_, kcal/mol		0	0.145	0.157
C–C bond length, Å		1.53	1.54	1.54
Torsion angle (terminal), °	C1–C2–C3–C4, C2–C3–C4–C5, C3–C4–C5–C6	−180.0	−179.7	59.9
Torsion angle (central), ° *trans-gauche-gauche-trans* part	C4–C5–C6–C7	−175.5	178.9	−177.8
	C5–C6–C7–C9	168.0	72.9	164.8
	C5–C6–C7–C8	−66.0	−161.4	−70.5
	C8–C7–C9–C10	66.0	70.5	161.5
	C6–C7–C9–C10	−168.0	−164.8	−72.8
	C7–C9–C10–C11	175.5	177.8	−178.9
Torsion angle (terminal), °	C9–C10–C11–C12, C10–C11–C12–C13, C11–C12–C13–C14, C12–C13–C14–C15, C13–C14–C15–C16	180.0	36.1	179.8
*ν*_C–C_, cm^−1^ (RSA, Å^4^/amu)		1136 (27.87)	1140 (8.16)	1140 (11.62)
			1146 (13.24)	1147 (10.80)

**Table 4:** Peak positions of *ν*_as_(CH_2_) and *ν*_as_(CH_3_) bands for the hydrogenated 1-hexene and 1-octene oligomers as well as for linear alkanes.

Samples	*ν*_as_(CH_2_) cm^−1^	*ν*_as_(CH_3_) cm^−1^	Samples	*ν*_as_(CH_2_), cm^−1^	*ν*_as_(CH_3_), cm^−1^
1-hexene oligomers			1-octene oligomers		
H2H, C_12_H_26_, *n* = 12, k = 2	2892	2960	O2H, C_16_H_34,_ *n* = 16, k = 2	2890	2960
H3H, C_18_H_38_, *n* = 18, k = 3	2895	2961	O3H, C_24_H_50_, *n* = 24, k = 3	2890	2961
H4H, C_24_H_50_, *n* = 24, k = 4	2894	2960	O4H, C_32_H_66_, *n* = 32, k = 4	2890	2960
H5H, C_30_H_62_, *n* = 30, k = 5	2895	2960	O5H, C_40_H_82_, *n* = 40, k = 5	2891	2960
Linear alkanes (k = 1)					
C_6_H_14_, *n* = 6	2895	2960	C_8_H_18_, *n* = 8	2894	2961
C_12_H_26_, *n* = 12	2891	2960	C_16_H_34_, *n* = 16 ^1^	2890	2961

^1^ Shemuratov Yu.V. et al. [24].

## Supplementary Materials

The following are available online at https://www.mdpi.com/2073-4360/12/9/2153/s1, Table S1: Orbital and auxiliary basis sets for program PRIRODA (4z.bas), Table S2: Atomic Cartesian coordinates for hydrogenated 1-hexene and 1-octene dimer conformations with the lowest energies, Table S3: The calculated total energy E and total energy E_0_ corrected for the zero point vibration energy (ZPVE) for hydrogenated 1-hexene and 1-octene dimer conformations, and the differences ΔE, ΔE_0_ between the energies of the corresponding and extended conformations, Table S4: The calculated Raman spectra for hydrogenated 1-hexene and 1-octene dimer conformations with the lowest energies. Video S1: In-phase.mp4, Video S2: Out-phase.mp4.

## Appendix A

**Table A1:** Wavenumbers of the symmetric C–C stretching mode, LAM, and D-LAM bands for linear alkanes ^1^.

Linear Alkane	Symmetric C–C Stretching Mode Wavenumbers, cm^−1^			LAM Wavenumbers, cm^−1^		D-LAM Wavenumbers, cm^−1^
	Observed Value	DFT calc. Value	*ν*_C–C_^2^	Observed Value	DFT Calc. Value	Observed Value
C_5_H_12_	1145	1142	1145	399	393	–
C_6_H_14_	1142	1139	1141	370	362	317/330
C_7_H_16_	1139	1137	1138	307	298	286
C_8_H_18_	1137	1135	1137	280	270	296
C_9_H_20_	1135	1134	1135	248 ^3^	237	267 ^3^
C_10_H_22_	1134	1133	1134	230	219	252
C_11_H_24_	1134	1133	1134	221	195	243
C_12_H_26_	1134	1132	1133	212	184	237
C_13_H_28_	1133	1132	1133	199	172	229
C_14_H_30_	1133	1131	1133	171	159	224
C_15_H_32_	1133	1131	1132	158 ^4^	149	223
C_16_H_34_	1133	1131	1132	150 ^5^	139	220 ^7^
C_17_H_36_	1132	1130	1132	141 ^4^	132	220 ^7^
C_18_H_38_	1130	1130	1132	133 ^6^	124	Lines are absent at room temperature
C_20_H_42_	1133	1130	1132	120	112	

^1^ All the observed wavenumbers are presented for room temperature unless otherwise specified. ^2^ $\nu_{C-C}=\sqrt{1130.5_{}^{2}+313^{2}{sin}^{2}\frac{\pi }{n}}$ (see the text). ^3^ Snyder R.G., Kim Y. [15]. ^4^ Measured at T = −105 °C, Olf H.G., Fanconi B. [34], the band is not observed at room temperature. ^5^ Measured at liquid nitrogen temperature, Kobayashi M., Koizumi H., Cho Y. [35]. ^6^ Parker S.F. et al. [30]. ^7^ The value is calculated by the relation $\nu_{D-LAM}\left(cm^{-1}\right)=\frac{5590}{n^{2}}+198.$ (see the text).
