# Supplementary material for: Raman Spectroscopy Study of Structurally Uniform Hydrogenated Oligomers of α-Olefins
Source: Polymers (Basel). 2020 Sep 21;12(9):2153. doi: 10.3390/polym12092153 (PMC7570275; doi:10.3390/polym12092153)
Supplement: Supplementary file 1 [file polymers-12-02153-s001.pdf]

Raman spectroscopy study of structurally uniform hydrogenated oligomers of α-olefins

Sergey M. Kuznetsov [1], Maria S. Iablochnikova [1,2], Elena A. Sagitova [1,*] Kirill A. Prokhorov [1,*] Gulnara Yu. Nikolaeva [1], Leila Yu. Ustynyuk [3], Pavel V. Ivchenko [3,4], Alexey A. Vinogradov [4], Alexander A. Vinogradov [4] and Ilya E Nifant'ev [3,4]

1 Prokhorov General Physics Institute of the Russian Academy of Sciences, Vavilov Str. 38, 119991 Moscow, Russia; kuznetsovsm@kapella.gpi.ru (S.M.K.), sagitova@kapella.gpi.ru (E.A.S.), cyrpro@gpi.ru (K.A.P.), homa1971@gmail.com (G.Yu.N.)
2 Moscow Institute of Physics & Technology, Institutskiy Per. 9, 141700 Dolgoprudnyi, Moscow Region, Russia; iablochnikova.ms@phystech.edu (M.S.I.)
3 Department of Chemistry, M.V. Lomonosov Moscow State University, Leninskie Gory 1-3, 119991 Moscow, Russia; leila_ust@mail.ru, inpv@org.chem.msu.ru (P.V.I.), inif@org.chem.msu.ru (I.E.N.)
4 A.V. Topchiev Institute of Petrochemical Synthesis of the Russian Academy of Sciences, Leninsky Pr. 29, 119991 Moscow; vinasora@ips.ac.ru. (A.A.V.)
* Correspondence: sagitova@kapella.gpi.ru; Tel.: +7-499-503-87-68

Supporting Information

Abstract

This document contains additional information on DFT calculations, including functional and basis sets, as well as some DFT calculation results:

- atomic Cartesian coordinates for hydrogenated 1-hexene and 1-octene dimer conformations;
- total energy E and total energy E_0 corrected for the zero point vibration energy (ZPVE) for hydrogenated 1-hexene and 1-octene dimer conformations;
- calculated Raman spectra of hydrogenated 1-hexene and 1-octene dimers in various conformations, and the differences ΔE, ΔE_0 between the energies of the corresponding and the extended conformations.

DFT calculation details

We used the OLYP functional, 4z.bas basis and the following input parameters: tolerance = $1{\cdot}10^{-7}$, trust = 0.01, convergence = $1{\cdot}10^{-8}$, accuracy = $1{\cdot}10^{-9}$, and field = 0.0001.

The PRIRODA software calculates the values of Raman scattering activity (RSA) for the jth transition using the following formula:

$$RSA_j = g_j\left(45\bar{\alpha}_j^2 + 7\bar{ß}_j^2\right), \qquad (1)$$

where g_j is the degeneracy of the jth transition, $\bar{\alpha}_j^2$ and $\bar{ß}_j^2$ are given by:

$$\bar{\alpha}_j^2 = \frac{1}{9}\left(\alpha_{xx}^j + \alpha_{yy}^j + \alpha_{zz}^j\right)^2,$$
$$ß_j^2 = \frac{1}{2}\left\{\left(\alpha_{xx}^j - \alpha_{yy}^j\right)^2 + \left(\alpha_{xx}^j - \alpha_{zz}^j\right)^2 + \left(\alpha_{yy}^j - \alpha_{zz}^j\right)^2 + 6\left[\left(\alpha_{xy}^j\right)^2 + \left(\alpha_{yz}^j\right)^2 + \left(\alpha_{xz}^j\right)^2\right]\right\}. \qquad (2)$$

Here α_{kl}^j is the derived polarizability tensor component.

The value of RSA is proportional to the sum of the integral intensities of a line for both parallel and crossed polarization directions of the scattered radiation relative to the polarization direction of the laser radiation. The integral Raman intensities (I_j) should be calculated as:

$$I_j = C\frac{\left(\nu_0 - \nu_j\right)^3}{1 - \exp\left[-\frac{hc\nu_j}{kT}\right]}\frac{1}{\nu_j} \cdot RSA_j, \qquad (3)$$

where ν_0 (cm^{-1}) is the wavenumber of the exciting radiation, ν_j (cm^{-1}) is the Raman wavenumber. C is a constant, which depends on the measurement conditions. In the case of registration of Raman spectra in the photon counting mode, for comparison of the calculated and experimental spectra, the calculated (or experimental) intensity should be corrected by the factor that equals to the ratio of the energies (wavenumbers) of the laser and scattered light [1].

Functional and basis sets

OLYP functional (Handy-Cohen exchange + LYP correlation [2]): is the non-hybrid functional, which gives the best result for calculations of the linear alkane Raman spectra as compared to a number of other hybrid or non-hybrid functionals: PBE, PBE0, mPBE, BLYP, B3LYP [3].

Orbital basis sets: the one-electron basis sets used are contracted sets of Gaussian functions with the angular part represented by real spherical harmonics. The largest angular momentum is specified in the compilation stage (by default, it corresponds to the quantum number $L \leq 6$). Orbital basis sets were used to solve the Kohn-Sham equations.

Auxiliary basis sets were used to expand the electron density for the fast evaluation of density functional exchange-correlation terms. This approach accelerates the computations without the noticeable loss of accuracy [4]. Auxiliary basis sets are uncontracted sets of Gaussian functions. The parameters of the orbital and auxiliary bases are presented in Table S1 with the permission of the author of the program PRIRODA Dr. D.N. Laikov [5].

Table S1. Orbital and auxiliary basis sets for program PRIRODA (4z.bas)

Element	Orbital basis set	Auxiliary basis set
**C**	6 17	6 22
	0 7 1.367023e+05 1.149857e-04	0 1 1.27377189e-01 1.0
	4.658354e+03 4.700194e-03	0 1 2.93612768e-01 1.0
	2.047269e+04 8.939779e-04	1 1 2.93612768e-01 1.0
	1.317591e+03 1.986559e-02	2 1 2.93612768e-01 1.0
	4.265356e+02 7.302752e-02	0 1 6.76796672e-01 1.0
	1.498533e+02 2.446272e-01	1 1 6.76796672e-01 1.0
	5.507877e+01 7.321017e-01	2 1 6.76796672e-01 1.0
	0 1 2.095411e+01 1.0	3 1 6.76796672e-01 1.0
	0 1 8.283238e+00 1.0	0 1 1.56006068e+00 1.0
	0 1 3.353267e+00 1.0	1 1 1.56006068e+00 1.0
	0 1 1.329332e+00 1.0	2 1 1.56006068e+00 1.0
	0 1 5.148633e-01 1.0	3 1 1.56006068e+00 1.0
	0 1 1.929573e-01 1.0	0 1 3.59604211e+00 1.0
	0 1 6.844859e-02 1.0	1 1 3.59604211e+00 1.0
	1 5 5.507877e+01 6.638955e-03	2 1 3.59604211e+00 1.0
	2.095411e+01 2.065215e-02	0 1 8.28911273e+00 1.0
	8.283238e+00 8.722283e-02	0 1 1.91069481e+01 1.0
	3.353267e+00 2.666486e-01	0 1 4.40427675e+01 1.0
	1.329332e+00 7.233516e-01	0 1 1.01521465e+02 1.0
	1 1 5.148633e-01 1.0	0 1 2.34013631e+02 1.0
	1 1 1.929573e-01 1.0	0 1 5.39416755e+02 1.0
	1 1 6.844859e-02 1.0	0 1 1.24339097e+03 1.0
	2 1 1.329332e+00 1.0	
	2 1 5.148633e-01 1.0	
	2 1 1.929573e-01 1.0	
	3 1 1.329332e+00 1.0	
	3 1 5.148633e-01 1.0	
**H**	1 9	1 11
	0 5 3.478070e+02 1.513033e-03	0 1 1.60858930e-01 1.0
	5.215071e+01 1.175012e-02	0 1 3.84471061e-01 1.0
	1.187790e+01 6.129405e-02	1 1 3.84471061e-01 1.0
	3.373735e+00 2.470924e-01	0 1 9.18929379e-01 1.0
	1.103423e+00 7.545899e-01	1 1 9.18929379e-01 1.0
	0 1 3.920238e-01 1.0	0 1 2.19634529e+00 1.0
	0 1 1.464123e-01 1.0	1 1 2.19634529e+00 1.0
	0 1 5.420483e-02 1.0	2 1 2.19634529e+00 1.0
	1 1 1.103423e+00 1.0	0 1 5.24951399e+00 1.0
	1 1 3.920238e-01 1.0	0 1 1.25469330e+01 1.0
	1 1 1.464123e-01 1.0	0 1 2.99885908e+01 1.0

	2 1 1.103423e+00 1.0<br>2 1 3.920238e-01 1.0	

Calculation results

Table S2. Atomic Cartesian coordinates for hydrogenated 1-hexene and 1-octene dimer conformations with the lowest energies. Please notice that all the following results are given without rounding with account of the accuracy of calculations

Extended conformation of 1-hexene dimer				Branched 1 conformation of 1-hexene dimer			
Atom	X coordinate	Y coordinate	Z coordinate	Atom	X coordinate	Y coordinate	Z coordinate
C	0.6379781	-1.2562705	-2.0144297	C	-0.5985901	3.5110285	0.3562141
C	-1.0105451	-1.2006569	-4.4224699	C	-0.0415654	5.1456410	2.6918663
H	-0.5723145	0.5219237	-5.5006903	C	-3.0988491	4.3585522	-0.8928791
C	-0.8036243	-3.5013425	-6.1745333	H	-1.5226425	4.9262478	4.1268804
H	-2.9940794	-1.0115891	-3.8397072	H	1.7623777	4.6574817	3.5758489
C	-2.6503774	-3.4270275	-8.4076974	H	0.0448397	7.1521463	2.1871267
H	1.1243830	-3.6697382	-6.9180488	H	-3.1702424	6.4329021	-0.8577493
H	-1.1472672	-5.2300790	-5.0731014	C	-3.5151881	3.4966441	-3.6298020
C	-2.4560775	-5.7148501	-10.1672751	H	-4.6910485	3.7232464	0.2829059
H	-4.5921201	-3.2858947	-7.6848101	C	-5.9824724	4.4745764	-4.7951275
H	-2.3257882	-1.6939292	-9.5051131	H	-1.9182218	4.1430717	-4.7921453
H	-2.8445227	-7.4819567	-9.1566667	H	-3.5043473	1.4263082	-3.7472440
H	-0.5633884	-5.8744357	-10.9960410	H	0.9233197	3.8595936	-1.0193156
H	-3.8105647	-5.5783859	-11.7280057	C	-6.3984963	3.6390972	-7.5331406
C	0.9106153	0.7501202	2.4931049	H	-7.5849263	3.8382155	-3.6374286
C	-0.1639711	0.8822321	-0.1969002	H	-6.0068106	6.5492225	-4.7046150
C	-0.1146743	2.8150967	4.2508523	H	-6.4691211	1.5740704	-7.6924623
H	0.3373163	2.7124404	-1.0457915	H	-4.8745020	4.3074961	-8.7682221
H	-2.2356094	0.8713601	-0.0638125	H	-8.1800016	4.3834591	-8.2822785
H	-2.1866300	2.6813136	4.3260931	C	2.0072220	-0.4872172	1.5934015
C	0.9418855	2.7067101	6.9462708	C	-0.5854206	0.6609022	0.9825545
H	0.3044610	4.6775068	3.4305927	C	1.9242003	-3.3420185	2.0899028
C	-0.0823095	4.7682621	8.7054729	H	-1.8783525	0.3257518	2.5756347
H	0.5257507	0.8445113	7.7695053	H	-1.3811310	-0.3878651	-0.6169402
H	3.0141420	2.8447740	6.8748069	H	1.1074045	-4.2995301	0.4367077
H	2.9785602	0.8852310	2.4483029	C	4.5056679	-4.5146211	2.6854642
H	0.4730332	-1.1110025	3.3072114	H	0.6245192	-3.7222418	3.6657691
C	0.9788730	4.6521710	11.3940400	C	4.4276310	-7.3684593	3.1785249
H	0.3338433	6.6316525	7.8882919	H	5.8087291	-4.1338379	1.1120451
H	-2.1532016	4.6322250	8.7835467	H	5.3235510	-3.5627919	4.3418329
H	3.0405261	4.8621021	11.4033577	H	2.8372316	0.4504138	3.2455075
H	0.5326816	2.8457152	12.3062845	H	3.3010072	-0.1128323	0.0111485
H	0.1930164	6.1593934	12.5771542	C	7.0100840	-8.5289229	3.7721381
C	3.4765553	-1.1795316	-2.6204507	H	3.1289058	-7.7541587	4.7526142
H	3.9801339	0.5810936	-3.5949561	H	3.6145620	-8.3250025	1.5240792
H	4.6300887	-1.2989245	-0.9095478	H	7.8513229	-7.6773797	5.4638088
H	4.0447062	-2.7515653	-3.8361951	H	8.3412830	-8.2525500	2.2081341
H	0.2485156	-3.0586550	-1.0486446	H	6.8580710	-10.5666395	4.1092400

Branched 2 conformation of 1-hexene dimer				Extended conformation of 1-octene dimer			
Atom	X coordinate	Y coordinate	Z coordinate	Atom	X coordinate	Y coordinate	Z coordinate
C	-1.4486855	1.1802518	-0.2208688	C	-7.2753667	5.0027195	0.8227684
C	-2.9091768	1.6548805	-0.2865204	H	-6.8849572	5.4684968	1.7355885
C	-0.7977024	1.1094602	-1.6194470	H	-7.1208075	5.7105577	-0.0004449
H	-3.5316703	0.9416343	-0.8416732	H	-8.3564403	4.8779085	0.9522391
H	-3.3351653	1.7516691	0.7184683	C	-6.5914767	3.6621014	0.5428923
H	-3.0062449	2.6288410	-0.7752510	H	-7.0386933	3.2091050	-0.3529925
H	-1.4376892	0.4989344	-2.2736513	H	-6.8050194	2.9689208	1.3685579
C	-0.5187386	2.4574404	-2.2977827	C	-5.0738745	3.7653878	0.3513358
H	0.1531196	0.5687840	-1.5451986	H	-4.8630570	4.4611418	-0.4737514
C	0.1785676	2.3288732	-3.6578504	H	-4.6291555	4.2202373	1.2483857
H	-1.4531244	3.0160340	-2.4349574	C	-4.3839154	2.4254598	0.0703387
H	0.1063106	3.0711025	-1.6327593	H	-4.8285961	1.9730535	-0.8277124
H	-0.8896658	1.9228362	0.3694661	H	-4.5983010	1.7301302	0.8945482
C	0.4689304	3.6701863	-4.3360285	C	-2.8650855	2.5304721	-0.1187648
H	1.1208033	1.7774486	-3.5314468	H	-2.4213838	2.9734716	0.7845964
H	-0.4417172	1.7143153	-4.3252568	H	-2.6613336	3.2352891	-0.9341832
H	1.1217490	4.2983405	-3.7180380	C	-2.1880888	1.1823099	-0.3994018
H	-0.4533481	4.2342996	-4.5198746	H	-2.5983797	0.7566888	-1.3271325
H	0.9664172	3.5273595	-5.3020426	H	-2.4729635	0.4849724	0.4001971
C	0.0299286	-0.5500911	1.0631984	C	-0.6464790	1.1986515	-0.4939562
C	-1.3582611	-0.1651655	0.5343463	H	-0.2719741	1.6985447	0.4133678
C	0.0488236	-1.8793600	1.8287732	C	-0.0960096	-0.2444198	-0.4768814
H	-2.0473684	-0.1267636	1.3888983	H	-0.3893205	-0.7496307	-1.4090415
H	-1.7379569	-0.9664617	-0.1165879	H	-0.6006310	-0.7921290	0.3306766
H	-0.3142733	-2.6803006	1.1688752	C	1.4183850	-0.3907646	-0.2772061
C	1.4271214	-2.2666682	2.3770533	H	1.7145733	0.1534898	0.6312344
H	-0.6681101	-1.8276020	2.6607073	H	1.9586763	0.0839879	-1.1054047
C	1.4476211	-3.5951580	3.1420249	C	1.8826706	-1.8482950	-0.1604102
H	2.1453299	-2.3202056	1.5460070	H	1.5780363	-2.3971447	-1.0630852
H	1.7916080	-1.4665854	3.0376033	H	1.3555377	-2.3295510	0.6757955
H	0.3932155	0.2496796	1.7245541	C	3.3944005	-2.0095121	0.0390695
H	0.7503825	-0.6101521	0.2379090	H	3.6999880	-1.4585705	0.9401638
C	2.8258246	-3.9768545	3.6880634	H	3.9218790	-1.5314644	-0.7988163
H	0.7315129	-3.5438824	3.9741128	C	3.8586508	-3.4659608	0.1592785
H	1.0858662	-4.3969860	2.4832317	H	3.5529733	-4.0172161	-0.7415790
H	3.2042175	-3.2193838	4.3849566	H	3.3318304	-3.9440889	0.9975624
H	3.5616796	-4.0794840	2.8814733	C	5.3701482	-3.6276651	0.3583067
H	2.7898692	-4.9312665	4.2255129	H	5.6773315	-3.0765423	1.2589600
				H	5.8983421	-3.1506923	-0.4800107
				C	5.8354244	-5.0834115	0.4795339
				H	5.5309245	-5.6361308	-0.4202465
				H	5.3103389	-5.5618525	1.3181067
				C	7.3453233	-5.2388976	0.6777774
				H	7.6831866	-4.7349172	1.5912657
				H	7.6287989	-6.2944123	0.7589580
				H	7.9056151	-4.8100073	-0.1617035
				C	-0.1455371	1.9989931	-1.7061669
				H	0.9468016	2.0428465	-1.7407013
				H	-0.4894438	1.5460111	-2.6450657
				H	-0.5035459	3.0323281	-1.6868467

Branched 1 conformation of 1-octene dimer				Branched 2 conformation of 1-octene dimer			
Atom	X coordinate	Y coordinate	Z coordinate	Atom	X coordinate	Y coordinate	Z coordinate
C	-2.3833310	0.0386172	0.6650864	C	-2.2938497	-0.8507326	-0.3985267
C	-3.1900452	0.6958150	1.7964701	C	-3.8179230	-1.0401617	-0.4617372
C	-3.1848454	0.0800543	-0.6557498	C	-1.7482544	-0.1161791	-1.6425915
H	-4.1594363	0.2010950	1.9245333	H	-4.1311391	-1.5779773	-1.3614975
H	-2.6706222	0.6459571	2.7580428	H	-4.1777603	-1.6123527	0.4008098
H	-3.3852537	1.7537211	1.5793304	H	-4.3365937	-0.0730133	-0.4559738
H	-3.1670549	1.1058527	-1.0524680	H	-0.7123445	0.1886234	-1.4533175
C	-2.7348046	-0.8991575	-1.7482391	C	-1.7710351	-0.9132876	-2.9540226
H	-4.2382400	-0.1300504	-0.4258915	H	-2.3144221	0.8177633	-1.7747060
C	-3.5849074	-0.8303157	-3.0233800	C	-1.1692137	-0.1549919	-4.1439636
H	-1.6860786	-0.7183620	-2.0152541	H	-1.2176840	-1.8526260	-2.8108726
H	-2.7728932	-1.9221054	-1.3468263	H	-2.7989604	-1.2027008	-3.2052071
H	-2.2502542	-1.0214311	0.9315567	H	-1.8401274	-1.8538030	-0.3782815
C	-3.1562927	-1.8169712	-4.1156926	C	-1.1775615	-0.9441597	-5.4583430
H	-4.6380632	-1.0131721	-2.7660454	H	-1.7183052	0.7869090	-4.2862211
H	-3.5474261	0.1918635	-3.4265605	H	-0.1356801	0.1331317	-3.9037516
H	-3.1935381	-2.8399935	-3.7140016	H	-2.2108078	-1.2316200	-5.7013855
H	-2.1033397	-1.6346431	-4.3752759	H	-0.6290861	-1.8868908	-5.3177434
C	0.0157569	0.3512683	1.6422267	C	-0.4268140	-0.2724864	1.3355533
C	-0.9786442	0.6625574	0.5152681	C	-1.8978077	-0.1439885	0.9176883
C	1.4090235	0.9514515	1.4148079	C	-0.1011737	0.4262732	2.6618508
H	-0.5342526	0.3227614	-0.4275669	H	-2.1674800	0.9200863	0.8469408
H	-1.0879023	1.7528542	0.4174483	H	-2.5169048	-0.5529283	1.7276945
H	1.3184819	2.0417711	1.3058481	H	-0.7519552	0.0243135	3.4515709
C	2.4145076	0.6399996	2.5297693	C	1.3622431	0.2894801	3.0985807
H	1.8081684	0.5861868	0.4576642	H	-0.3541012	1.4929755	2.5785656
C	3.8077081	1.2387546	2.3023877	C	1.6884414	0.9884355	4.4238300
H	2.0168708	1.0067890	3.4869420	H	1.6154009	-0.7771885	3.1820853
H	2.5045934	-0.4502496	2.6397944	H	2.0133771	0.6921044	2.3094144
H	0.1080272	-0.7394947	1.7461184	H	0.2290549	0.1328684	0.5549520
H	-0.3714198	0.7151201	2.6020298	H	-0.1729395	-1.3389698	1.4204005
C	4.8132860	0.9275627	3.4170086	C	3.1515131	0.8517506	4.8610280
H	4.2058193	0.8718236	1.3454393	H	1.4356082	2.0551911	4.3404013
H	3.7178706	2.3290391	2.1924616	H	1.0375386	0.5859586	5.2132538
H	4.9042037	-0.1627058	3.5280011	H	3.8037016	1.2542170	4.0723897
H	4.4165920	1.2945700	4.3747180	H	3.4056707	-0.2148299	4.9452068
C	-4.0046375	-1.7490843	-5.3909139	C	-0.5758215	-0.1880920	-6.6486114
H	-5.0574371	-1.9326522	-5.1345366	H	-1.1234309	0.7538517	-6.7924715
H	-3.9675379	-0.7279503	-5.7955329	H	0.4577317	0.0987337	-6.4091543
C	-3.5727565	-2.7363676	-6.4781379	C	-0.5866955	-0.9806259	-7.9582268
H	-3.6364297	-3.7729359	-6.1258866	H	-0.1475238	-0.4002369	-8.7775784
H	-4.2071709	-2.6504317	-7.3676240	H	-0.0131548	-1.9109551	-7.8679710
H	-2.5373532	-2.5581008	-6.7924178	H	-1.6075352	-1.2502441	-8.2545295
C	6.2067404	1.5253756	3.1905738	C	3.4789429	1.5502065	6.1859098
H	6.6062271	1.1586035	2.2346889	H	2.8295275	1.1484375	6.9762641
H	6.1187785	2.6153704	3.0810204	H	3.2274796	2.6169009	6.1037477
C	7.2065343	1.2116048	4.3064707	C	4.9409518	1.4104267	6.6174917
H	7.3543914	0.1308601	4.4193188	H	5.1257259	1.9247936	7.5674088
H	8.1854989	1.6589785	4.1002038	H	5.2199234	0.3584043	6.7517713
H	6.8628882	1.5998967	5.2727718	H	5.6212533	1.8392054	5.8718752

Table S3. The calculated total energy E and total energy E_0 corrected for the zero point vibration energy (ZPVE) for hydrogenated 1-hexene and 1-octene dimer conformations, and the differences ΔE, ΔE_0 between the energies of the corresponding and extended conformations. Please notice that all the following results are given without rounding with account of the accuracy of calculations

**Properties**	**Conformations of 1-hexene dimer**		
	**Extended**	**Branched 1**	**Branched 2**
**E, kcal/mol**	-296755.504979	-296755.392654	-296755.392654
**ΔE, kcal/mol**	0	0.112324	0.112324
**E_0, kcal/mol**	-296534.273190	-296534.129491	-296534.128236
**ΔE_0, kcal/mol**	0	0.143700	0.144955

**Properties**	**Conformations of 1-octene dimer**		
	**Extended**	**Branched 1**	**Branched 2**
**E, kcal/mol**	-395423.231470	-395423.123538	-395423.124793
**ΔE, kcal/mol**	0	0.107932	0.106677
**E_0, kcal/mol**	-395131.933170	-395131.788216	-395131.776293
**ΔE_0, kcal/mol**	0	0.144955	0.156877

Table S4. The calculated Raman spectra for hydrogenated 1-hexene and 1-octene dimer conformations with the lowest energies. Please notice that all the following results are given without rounding with account of the accuracy of calculations

**Extended conformation of 1-hexene dimer**		**Branched 1 conformation of 1-hexene dimer**		**Branched 2 conformation of 1-hexene dimer**	
**Wavenumber, cm^{-1}**	**RSA*, $Å^4$/amu**	**Wavenumber, cm^{-1}**	**RSA*, $Å^4$/amu**	**Wavenumber, cm^{-1}**	**RSA*, $Å^4$/amu**
27.38	0.203	25.72	0.599	28.78	0.641
35.01	0.178	33.01	0.746	36.38	0.596
46.39	0.244	47.13	0.113	39.21	0.189
61.14	0.182	61.10	0.104	62.37	0.068
71.70	0.198	78.73	0.093	85.23	0.016
101.80	0.061	86.29	0.061	100.75	0.142
116.14	0.075	113.37	0.033	111.53	0.016
119.87	0.035	134.10	0.083	127.04	0.046
135.47	0.038	140.26	0.067	137.35	0.032
149.53	0.064	154.29	0.050	156.87	0.073
194.85	4.762	196.65	0.375	184.41	0.458
210.24	2.739	216.33	2.980	223.36	4.513
230.18	0.107	230.66	1.436	238.11	0.450
243.86	0.027	244.24	0.032	244.05	0.007
244.83	0.035	244.70	0.409	244.72	0.079
297.53	0.514	289.55	0.897	294.96	1.271
306.99	0.285	322.89	3.910	314.18	2.121
394.76	0.221	373.80	0.466	370.77	2.128
411.81	0.633	406.72	1.121	416.70	0.811
424.53	0.373	465.64	0.433	455.56	0.785
506.00	0.134	481.50	0.382	484.47	0.457
520.29	0.543	511.19	0.691	513.73	0.390
718.53	0.023	717.76	0.078	718.23	0.044
722.21	0.028	720.46	0.044	720.22	0.118
729.87	0.150	727.84	0.228	728.70	0.125
772.10	0.257	772.45	0.514	771.34	0.850
777.84	0.703	776.20	0.403	776.69	0.194
835.55	2.587	837.12	1.984	836.73	1.561
869.33	0.113	867.72	0.263	871.23	1.268
885.76	5.872	885.99	3.353	885.60	3.528
892.52	6.171	891.97	9.403	893.21	8.078
901.44	0.503	904.79	1.485	900.69	0.552
923.46	2.751	930.12	2.796	925.83	4.414
958.62	0.268	956.37	0.580	966.62	0.619
995.60	0.871	993.25	0.704	993.49	2.021
1004.71	1.846	1015.29	1.852	1006.93	0.333
1012.84	1.063	1017.31	2.837	1012.61	0.204
1029.23	0.501	1020.13	0.272	1027.75	1.132
1047.18	2.588	1036.15	1.044	1036.53	3.014
1055.09	2.324	1052.87	1.636	1053.74	2.497
1059.57	7.059	1059.91	6.621	1057.95	5.727
1063.82	4.362	1061.78	7.138	1062.03	7.857
1068.20	1.401	1077.09	1.154	1082.16	1.526
1101.65	1.601	1099.24	3.062	1096.16	1.923

1139.24	17.260	1142.58	6.285	1141.84	8.586
1155.16	0.884	1149.09	8.241	1150.09	6.370
1172.74	5.899	1171.17	5.300	1171.44	5.379
1201.29	0.338	1202.26	1.155	1199.13	1.168
1220.88	0.732	1218.61	0.759	1228.24	1.020
1234.36	0.734	1238.28	0.335	1232.47	0.304
1253.42	0.862	1256.79	0.275	1251.80	0.690
1265.75	0.745	1263.75	0.897	1269.76	0.231
1285.71	0.980	1283.43	0.667	1283.48	1.046
1296.98	1.431	1299.47	2.303	1296.15	2.939
1301.25	7.080	1304.16	6.532	1304.89	3.863
1311.47	14.475	1311.85	7.131	1312.43	6.603
1314.42	4.839	1314.26	9.090	1314.41	13.069
1317.10	0.526	1316.59	2.073	1317.36	0.269
1319.14	0.566	1323.06	0.648	1324.30	1.113
1343.46	0.694	1333.79	3.580	1332.45	3.488
1352.71	1.600	1352.67	2.652	1353.59	2.082
1354.12	2.257	1355.91	0.370	1354.65	1.212
1363.75	0.653	1366.33	0.687	1366.23	0.423
1368.11	0.554	1367.77	0.808	1368.28	0.800
1370.95	0.357	1369.98	0.289	1370.11	0.263
1375.84	0.136	1375.16	0.126	1376.24	0.159
1377.53	0.209	1377.53	0.189	1377.49	0.302
1381.89	0.415	1380.00	0.782	1379.26	0.467
1441.88	0.807	1445.85	6.953	1445.99	7.210
1450.38	3.984	1450.38	13.941	1450.36	12.157
1451.00	9.868	1450.83	3.096	1451.37	4.496
1452.81	29.584	1453.08	8.768	1452.66	9.882
1454.08	15.945	1453.74	23.166	1453.77	19.454
1461.63	1.551	1461.07	1.222	1461.32	4.229
1464.99	4.775	1463.87	0.680	1464.07	4.641
1465.42	1.908	1465.71	4.712	1465.75	4.337
1465.73	4.926	1465.77	4.531	1465.78	4.553
1465.86	3.528	1468.17	6.546	1467.19	3.243
1471.85	1.842	1470.17	4.208	1471.92	2.658
1477.09	0.788	1474.00	1.156	1472.62	1.884
1479.18	1.043	1477.74	0.578	1478.21	1.079
1481.81	1.376	1479.83	0.464	1479.80	0.378
2941.96	105.781	2945.56	87.018	2945.65	87.248
2953.31	23.593	2955.00	126.987	2954.75	72.843
2955.86	56.279	2957.12	7.589	2957.65	21.399
2958.00	2.072	2958.14	5.629	2958.70	30.525
2960.15	404.865	2961.76	301.645	2961.29	361.054
2961.83	19.088	2962.81	94.587	2963.24	11.039
2967.16	152.703	2968.24	108.431	2967.30	151.625
2971.17	14.031	2971.44	32.226	2972.84	13.905
2974.18	130.790	2975.07	50.185	2974.19	61.140
2977.76	59.299	2977.92	137.046	2978.03	14.179
2978.01	355.359	2978.09	323.012	2978.15	445.628
2979.22	142.264	2979.26	148.043	2979.13	155.189
2984.40	178.650	2981.03	231.147	2981.01	211.694
2987.93	16.396	2988.17	130.686	2988.71	17.326

2990.72	99.601	2990.46	32.809	2990.60	150.311
2992.40	100.637	2995.64	10.696	2994.75	74.004
2998.18	60.231	3004.12	131.834	3006.03	55.685
3010.49	14.790	3013.77	12.082	3010.29	22.079
3022.62	6.589	3021.66	7.949	3024.82	7.213
3028.72	5.312	3032.30	11.757	3030.32	23.573
3048.03	23.436	3046.35	98.793	3046.52	87.824
3048.14	40.865	3048.10	32.059	3048.09	31.800
3050.32	112.463	3048.22	16.716	3048.17	21.128
3050.67	114.262	3050.41	111.628	3050.34	103.324
3057.25	95.571	3050.54	110.440	3050.56	112.407
3073.70	59.355	3065.57	78.606	3065.27	87.290

**Extended conformation of 1-octene dimer**		**Branched 1 conformation of 1-octene dimer**		**Branched 2 conformation of 1-octene dimer**	
**Wavenumber, cm^{-1}**	**RSA*, $Å^4$/amu**	**Wavenumber, cm^{-1}**	**RSA*, $Å^4$/amu**	**Wavenumber, cm^{-1}**	**RSA*, $Å^4$/amu**
15.65	0.190	15.32	0.783	15.90	0.803
24.34	0.367	19.75	0.794	24.46	0.724
29.08	0.159	31.90	0.175	26.84	0.265
38.26	0.271	41.43	0.149	40.25	0.068
46.22	0.263	49.40	0.131	50.09	0.038
65.13	0.141	54.04	0.062	64.57	0.121
69.46	0.011	79.67	0.012	79.75	0.050
82.70	0.046	84.04	0.042	81.99	0.085
98.83	0.067	99.50	0.090	88.43	0.016
113.23	0.195	111.62	0.073	113.18	0.107
122.40	0.252	118.27	0.052	126.78	0.072
128.26	0.037	136.19	0.088	134.06	0.073
141.53	0.162	141.90	0.022	141.38	0.016
148.91	1.604	151.21	0.610	152.12	0.177
155.32	3.239	156.15	0.020	156.66	0.275
157.10	1.581	170.16	2.347	179.13	4.097
191.09	0.183	207.21	3.785	202.46	1.078
231.00	0.119	227.97	0.849	215.98	1.104
243.63	0.152	240.33	0.333	243.07	1.084
244.34	0.015	244.34	0.007	244.42	0.003
244.58	0.016	244.57	0.022	244.45	0.047
270.68	0.143	264.42	1.385	271.63	2.042
339.50	0.230	335.85	1.424	321.19	1.750
342.72	0.588	347.25	1.878	376.17	0.896
410.32	1.569	398.27	0.057	378.70	0.435
415.71	0.777	412.39	1.248	420.84	0.609
434.05	0.182	471.61	0.047	451.98	0.331
472.48	0.038	472.37	0.107	475.92	0.048
513.00	0.031	487.76	0.249	504.96	0.580
534.59	0.100	528.48	0.353	522.12	0.329
717.64	0.012	717.57	0.086	717.92	0.066
718.93	0.005	718.44	0.016	718.49	0.027
720.30	0.053	719.84	0.088	720.14	0.058
729.04	0.086	728.72	0.167	728.28	0.253

736.85	0.128	735.68	0.142	736.44	0.059
775.49	0.047	775.73	0.515	775.21	0.479
779.24	0.705	778.19	0.390	778.53	0.358
830.78	2.689	832.24	2.443	831.28	2.169
851.25	0.982	850.40	1.119	853.97	1.716
871.71	0.327	873.42	0.482	869.91	0.734
884.72	9.937	885.51	6.514	885.40	7.266
887.41	0.457	887.50	3.593	887.91	3.052
916.78	1.081	919.48	1.107	916.99	2.447
925.67	2.325	925.72	4.243	933.39	1.309
957.62	0.249	966.41	0.715	955.98	0.659
980.42	0.545	980.81	0.904	981.47	0.385
990.02	0.693	984.76	0.850	989.12	1.005
996.38	0.868	999.21	0.988	1001.49	0.711
1007.63	2.188	1005.49	0.837	1011.92	3.819
1020.22	0.328	1019.11	0.930	1016.58	2.485
1029.35	1.229	1026.38	0.973	1022.92	0.859
1031.33	1.138	1040.34	1.425	1037.59	0.363
1046.16	3.917	1045.33	1.951	1040.13	0.346
1057.37	0.497	1048.90	3.216	1054.59	1.205
1058.97	6.919	1054.44	1.644	1055.03	3.552
1061.46	2.303	1060.36	3.711	1059.94	3.254
1063.17	4.454	1062.07	9.976	1061.90	8.553
1064.08	3.324	1063.14	4.587	1062.79	6.534
1086.24	1.642	1095.91	1.246	1099.97	2.061
1112.65	1.781	1110.13	5.420	1107.18	3.811
1136.01	27.866	1140.59	8.156	1139.58	11.620
1154.67	0.921	1146.14	13.242	1146.86	10.797
1172.82	7.849	1171.19	6.701	1171.36	6.650
1191.07	0.379	1191.88	1.357	1189.53	1.592
1204.70	0.861	1201.70	1.167	1207.08	1.250
1214.74	0.388	1219.89	0.107	1217.82	0.056
1229.18	0.317	1231.36	0.187	1227.07	0.280
1239.69	0.429	1238.43	0.744	1241.24	0.340
1255.76	0.633	1254.44	0.599	1259.44	0.262
1265.19	0.645	1267.97	0.077	1262.40	1.252
1276.85	1.170	1278.70	2.575	1276.87	0.631
1284.51	2.400	1284.80	0.180	1289.57	0.767
1300.77	5.715	1296.51	2.063	1296.54	2.865
1303.16	5.777	1306.70	5.099	1303.80	2.015
1304.90	0.602	1307.71	16.859	1307.48	19.933
1307.66	16.809	1308.53	4.784	1308.81	5.586
1312.64	8.352	1313.05	8.304	1312.95	6.717
1318.42	0.518	1318.35	0.299	1318.63	0.020
1319.28	0.327	1319.60	0.625	1319.53	1.388
1320.30	0.688	1319.85	0.426	1320.12	0.360
1323.42	0.662	1326.10	0.644	1327.85	1.365
1343.89	0.540	1335.63	3.234	1334.68	2.987
1351.18	1.733	1351.83	2.221	1352.28	0.677
1353.91	2.621	1353.89	1.024	1352.98	2.430
1362.15	1.219	1364.57	1.116	1365.76	1.543
1367.69	0.357	1367.63	1.155	1367.68	0.852

1368.10	0.350	1368.49	0.297	1368.44	0.416
1369.03	0.385	1370.78	0.149	1370.07	0.101
1372.20	0.329	1371.02	0.081	1371.47	0.118
1377.26	0.329	1377.41	0.216	1376.82	0.384
1377.67	0.276	1377.72	0.262	1377.66	0.286
1381.00	0.307	1378.64	0.546	1379.16	0.344
1441.12	0.756	1445.66	5.614	1445.75	5.015
1448.97	1.040	1448.96	1.049	1449.01	1.549
1449.19	0.668	1449.30	2.446	1449.48	2.754
1450.53	4.145	1450.61	19.954	1450.54	18.739
1451.15	12.706	1451.48	9.530	1451.32	8.369
1452.64	59.383	1452.70	28.779	1453.01	27.886
1453.73	13.642	1453.83	17.413	1453.82	22.851
1459.31	6.719	1459.07	5.519	1459.39	2.141
1461.75	0.347	1461.44	3.243	1461.22	1.864
1465.09	2.281	1465.68	5.225	1465.66	4.711
1465.68	4.819	1465.71	4.439	1465.68	4.659
1465.72	4.907	1466.08	2.338	1465.90	5.977
1467.57	0.383	1467.61	1.456	1468.81	0.740
1471.77	1.623	1471.81	4.215	1470.15	4.214
1474.91	1.269	1472.60	2.024	1473.99	1.136
1478.96	0.910	1476.00	0.481	1475.64	1.086
1480.29	0.801	1479.88	0.393	1480.02	0.544
1481.86	1.029	1480.87	0.305	1480.85	0.265
2941.74	103.689	2945.53	84.858	2945.73	85.467
2952.84	7.508	2954.25	12.275	2954.14	13.185
2954.50	3.602	2954.88	43.758	2955.16	91.840
2955.89	5.204	2955.96	18.193	2956.01	25.113
2956.01	168.108	2957.50	161.247	2957.08	125.130
2957.97	38.708	2958.27	238.100	2958.03	43.786
2958.84	52.552	2959.31	12.379	2959.75	194.545
2960.99	581.894	2962.23	217.391	2962.59	212.106
2963.27	100.541	2964.54	235.502	2963.70	230.753
2967.08	41.500	2967.30	65.638	2968.23	33.416
2969.96	127.596	2970.89	96.541	2969.97	134.403
2973.28	7.468	2973.49	21.594	2974.21	10.401
2975.08	100.498	2975.76	31.262	2975.26	43.173
2977.95	217.618	2978.03	214.995	2978.02	140.481
2978.06	6.929	2978.10	64.819	2978.10	309.714
2978.13	412.489	2978.16	413.911	2978.12	242.390
2979.21	147.525	2979.09	159.137	2979.21	145.950
2981.49	0.916	2980.87	177.150	2980.90	224.044
2984.37	192.894	2981.57	54.392	2981.49	14.145
2988.17	5.053	2988.73	18.714	2988.94	77.210
2990.63	85.663	2990.40	73.205	2990.35	30.313
2991.63	21.321	2994.64	64.512	2993.85	12.962
2997.31	98.942	3001.23	42.328	3003.68	128.736
3004.90	27.632	3008.53	35.510	3004.86	15.979
3010.44	7.881	3010.61	54.220	3013.67	11.828
3018.27	7.216	3019.74	3.010	3017.96	12.279
3026.94	0.919	3026.12	2.218	3026.96	1.061
3030.10	2.872	3032.62	10.454	3032.50	14.057

3048.05	28.423	3046.40	90.021	3046.35	85.383
3048.09	38.684	3048.06	25.132	3048.04	28.828
3050.46	110.138	3048.09	35.043	3048.09	33.270
3050.49	119.227	3050.47	113.065	3050.52	111.212
3057.19	103.776	3050.54	114.950	3050.57	114.708
3073.63	60.157	3065.16	84.716	3065.15	88.207

* RSA is the Raman scattering activity, see formula (1).

References

[1] Rudolph W.W., Irmer G., *Appl. Spectrosc.*, 2007, **61**, 1312-1327, DOI: 10.1366/000370207783292037

[2] Baker J., Pulay P., *J. Chem. Phys.*, 2002, **117**, 1441-1449, DOI: 10.1063/1.1485723

[3] Kuznetsov S.M., Novikov V.S., Sagitova E.A., Ustynyuk L.Yu., Glikin A.A., Prokhorov K.A., Nikolaeva G.Yu., Pashinin P.P., *Laser Phys.*, 2019, **29**, 085701 (15 pp.), Supplementary material – 18 pp., DOI: 10.1088/1555-6611/ab2908

[4] Laikov D.N., *Chem. Phys. Lett.*, 1997, **281**, 151–156, DOI: 10.1016/S0009-2614(97)01206-2

[5] Laikov D.N., *Chem. Phys. Lett.*, 2005, **416**, 116–120, DOI: 10.1016/j.cplett.2005.09.046
